# Efficacy of Systemic Chemotherapy in Patients With Low-grade Mucinous Appendiceal Adenocarcinoma: A Randomized Crossover Trial

**DOI:** 10.1001/jamanetworkopen.2023.16161

**Published:** 2023-06-01

**Authors:** John Paul Shen, Abdelrahman M. Yousef, Fadl A. Zeineddine, Mohammad A. Zeineddine, Rebecca S. Tidwell, Karen A. Beaty, Lisa C. Scofield, Safia Rafeeq, Nicholas Hornstein, Elizabeth Lano, Cathy Eng, Aurelio Matamoros, Wai Chin Foo, Abhineet Uppal, Christopher Scally, Paul Mansfield, Melissa Taggart, Kanwal P. Raghav, Michael J. Overman, Keith Fournier

**Affiliations:** 1 Department of Gastrointestinal Medical Oncology, University of Texas MD Anderson Cancer Center, Houston; 2 Department of Internal Medicine, Houston Methodist Hospital, Houston, Texas; 3 Department of Biostatistics, University of Texas MD Anderson Cancer Center, Houston; 4 Department of Surgical Oncology, University of Texas MD Anderson Cancer Center, Houston; 5 Department of Radiology, University of Texas MD Anderson Cancer Center, Houston; 6 Department of Pathology, University of Texas MD Anderson Cancer Center, Houston; 7 Department of Medical Oncology, Vanderbilt University, Nashville, Tennessee

## Abstract

**Importance:**

Appendiceal adenocarcinoma is a rare tumor, and given the inherent difficulties in performing prospective trials in such a rare disease, there are currently minimal high-quality data to guide treatment decisions, highlighting the need for more preclinical and clinical investigation for this disease.

**Objective:**

To prospectively evaluate the effectiveness of fluoropyrimidine-based systemic chemotherapy in patients with inoperable low-grade mucinous appendiceal adenocarcinoma.

**Design, Setting, and Participants:**

This open-label randomized crossover trial recruited patients at a single tertiary care comprehensive cancer center from September 2013 to January 2021. The data collection cutoff was May 2022. Enrollment of up to 30 patients was planned. Eligible patients had histological evidence of a metastatic low-grade mucinous appendiceal adenocarcinoma, with radiographic imaging demonstrating the presence of mucinous peritoneal carcinomatosis and were not considered candidates for complete cytoreductive surgery. Key exclusion criteria were concurrent or recent investigational therapy, evidence of bowel obstruction, and use of total parenteral nutrition. Data were analyzed from November 2021 to May 2022.

**Interventions:**

Patients were randomized to either 6 months observation followed by 6 months of chemotherapy, or initial chemotherapy followed by observation.

**Main Outcomes and Measures:**

The primary end point was the percentage difference in tumor growth in treatment and observation groups. Key secondary end points included patient-reported outcomes in the chemotherapy and observation periods, objective response rate, rate of bowel complications, and differences in overall survival (OS).

**Results:**

A total of 24 patients were enrolled, with median (range) age of 63 (38 to 82) years, and equal proportion of men and women (eg, 12 men [50%]); all patients had ECOG performance status of 0 or 1. A total of 11 patients were randomized to receive chemotherapy first, and 13 patients were randomized to receive observation first. Most patients (15 patients [63%]) were treated with either fluorouracil or capecitabine as single agent; 3 patients (13%) received doublet chemotherapy (leucovorin calcium [folinic acid], fluorouracil, and oxaliplatin or folinic acid, fluorouracil, and irinotecan hydrochloride), and bevacizumab was added to cytotoxic chemotherapy for 5 patients (21%). Fifteen patients were available to evaluate the primary end point of difference in tumor growth during treatment and observation periods. Tumor growth while receiving chemotherapy increased 8.4% (95% CI, 1.5% to 15.3%) from baseline but was not significantly different than tumor growth during observation (4.0%; 95% CI, −0.1% to 8.0%; *P* = .26). Of 18 patients who received any chemotherapy, none had an objective response (14 patients [77.8%] had stable disease; 4 patients [22.2%] had progressive disease). Median (range) OS was 53.2 (8.1 to 95.5) months, and there was no significant difference in OS between the observation-first group (76.0 [8.6 to 95.5] months) and the treatment-first group (53.2 [8.1 to 64.1] months; hazard ratio, 0.64; 95% CI, 0.16-2.55; *P* = .48). Patient-reported quality-of-life metrics identified that during treatment, patients experienced significantly worse fatigue (mean [SD] score, 18.5 [18.6] vs 28.9 [21.3]; *P* = .02), peripheral neuropathy (mean [SD] score, 6.67 [12.28] vs 38.89 [34.88]; *P* = .01), and financial difficulty (mean [SD] score, 8.9 [15.2] vs 28.9 [33.0]; *P* = .001) compared with during observation.

**Conclusions and Relevance:**

In this prospective randomized crossover trial of systemic chemotherapy in patients with low-grade mucinous appendiceal adenocarcinoma, patients did not derive clinical benefit from fluorouracil-based chemotherapy, given there were no objective responses, no difference in OS when treatment was delayed 6 months, and no difference in the rate of tumor growth while receiving chemotherapy.

**Trial Registration:**

ClinicalTrials.gov Identifier: NCT01946854

## Key Points

**Question:**

Is fluoropyrimidine-based systemic chemotherapy effective in treating patients with inoperable low-grade mucinous appendiceal adenocarcinoma?

**Findings:**

In this randomized crossover trial that included 24 patients, there was no significant difference in tumor growth between treatment and observation time periods.

**Meaning:**

These findings suggest that patients with low-grade mucinous appendiceal adenocarcinoma did not derive clinically meaningful benefit from systemic fluoropyrimidine-based chemotherapy.

## Teaser

This randomized crossover trial evaluates the effectiveness of fluoropyrimidine-based systemic chemotherapy in patients with inoperable low-grade mucinous appendiceal adenocarcinoma.

## Introduction

Appendiceal adenocarcinoma (AA) is both a rare and heterogenous disease, with marked contrast in the natural history of low-grade vs high-grade tumors (5-year overall survival [OS], 68% vs 7%).^1,2,3,4^ The rarity of AA has made it difficult to study with traditional prospective trials; thus, there has been a critical lack of data regarding the responsiveness of appendiceal tumors to chemotherapy. Traditionally, AA has been treated with chemotherapy approved for the treatment of colorectal cancer (CRC), although the evidence to support this practice is primarily anecdotal or in the form of small case series.^5,6^ In the United States, current National Comprehensive Cancer Network guidelines continue to suggest that appendiceal cancer be treated similarly to CRC.^7^ However, low-grade mucinous AAs are known to be distinctly different from CRC in terms of natural history, with lack of lymph node and hematogenous spread, indolent growth, and limited cytological atypia.^8,9,10^ There is also an evolving body of molecular data that have identified clear molecular differences between AA and CRC.^2,11,12,13^ Finally, while the literature on chemotherapy response in AA is limited, the few existing reports suggest limited clinical activity of systemic chemotherapy, especially in patients with mucinous histology and well-differentiated (low-grade) tumors.^5,14,15,16,17,18^

Histologically low-grade appendiceal tumors are generally hypocellular with abundant mucin and pushing, as opposed to infiltrating, margins.^19^ These tumors are primarily treated with cytoreductive surgery (CRS) that is often followed by hyperthermic intraperitoneal chemotherapy (HIPEC); this is currently considered standard-of-care treatment.^14,20,21,22,23,24,25^ However, despite an absence of prospective data suggesting that patients with low-grade AA benefit from systemic chemotherapy, it is common practice that patients with inoperable, low-grade AA are treated with systemic chemotherapy.^18,26,27,28^ The cytotoxic effects of most traditional chemotherapy drugs, such as nucleoside analogs (eg, fluoropyrimidine), are dependent on the rate of cell division (cell cycle phase–specific chemotherapy), which is why it has been hypothesized that the slow growth of this disease would result in intrinsic resistance.^5,29^ Retrospective studies of systemic chemotherapy in low-grade AA have suggested a lack of benefit; these negative results are consistent with our institution’s experience with these low-grade tumors.^14,15,16,17,18^ Therefore, we aimed to conduct a prospective, randomized crossover trial to objectively evaluate the effectiveness of systemic chemotherapy in low-grade mucinous AA, the first such study to our knowledge.

In nearly all patients, metastatic spread of AA is limited to the peritoneal cavity, causing the clinical syndrome pseudomyxoma peritonei (PMP).^30,31,32^ Mucinous peritoneal disease is difficult to measure with traditional cross-sectional imaging, since it frequently exists as a contiguous, erratically shaped area in the peritoneal cavity (eFigure 1 in Supplement 1). In addition, current Response Evaluation Criteria in Solid Tumors (RECIST) criteria do not consider mucinous or cystic disease to be measurable. For these reasons, standard RECIST criteria are poorly applicable to AA.^33^ Moreover, AA is a slowly progressive disease, and classically defined thresholds for determining changes in disease extent (typically 20% increase) may take years to occur. Thus, determining systemic chemotherapy benefit through standard outcome measures, such as traditional RECIST response rate and time to disease progression, is not practical in this tumor type. To better quantify peritoneal disease burden, the modified peritoneal RECIST (mpRECIST), which measures up to 5 areas of mucinous disease in the abdominal cavity, was developed. The general mpRECIST guidelines for tumor evaluation follow the structure established by RECIST version 1.1, with 2 fundamental differences: up to 5 lesions in the peritoneal cavity are assessed and mucinous lesions are considered measurable disease.

## Methods

From September 2013 to January 2021, we conducted a prospective randomized crossover trial in patients with low-grade mucinous AA. The University of Texas MD Anderson Cancer Center (MDACC) institutional review board approved the trial protocol in Supplement 2. Patients provided written informed consent. We followed the Consolidated Standards of Reporting Trials (CONSORT) reporting guideline.

### Patients

Eligible patients had histological evidence of a metastatic low-grade (defined as well- or well-to-moderate–differentiated) mucinous AA, with radiographic images demonstrating the presence of PMP, and were not considered candidates for complete CRS. Surgical candidacy was determined by consultation with peritoneal surface malignancy surgeons at MDACC, in coordination with our multidisciplinary peritoneal surface malignancy conference. Criteria for determining nonresectability were: medical comorbidities presenting high surgical risk; tumor bulk and location, such as encasement of the liver hilum or extensive small bowel involvement that would preclude the possibility of obtaining a complete cytoreduction (completeness of cytoreduction score of 0 or 1); or prior CRS that was unsuccessful. Patients were required to have Eastern Cooperative Oncology Group (ECOG) Performance Status of 0 to 2, be aged at least 18 years, and have adequate bone marrow function (hemoglobin, ≥9.0 g/dL [to convert to grams per liter, multiply by 10]; platelets, ≥75 cells × 10^3^/μL [to convert to ×10^9^/L, multiply by 1]; absolute neutrophil count, ≥1000/μL). Key exclusion criteria were concurrent uncontrolled medical illness that was deemed by the investigator to have the potential to interfere with the delivery of chemotherapy, concurrent or recent investigational therapy, evidence of a bowel obstruction, use of total parenteral nutrition, and concurrent nonappendiceal metastatic cancer.

### Study Design and Treatment

**Figure 1.**
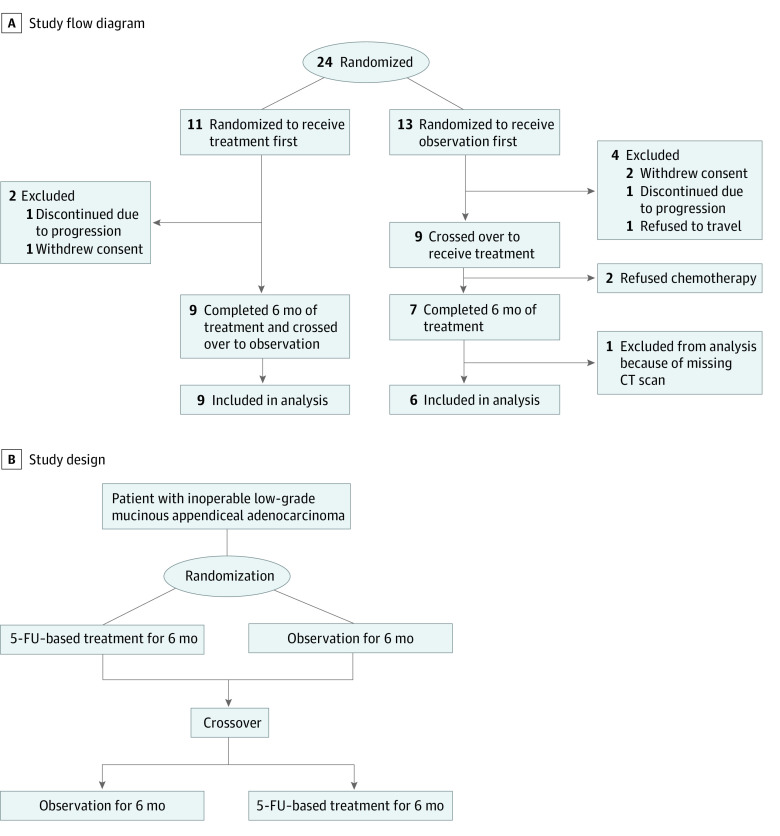
Participant Recruitment Flowchart Total study duration was 12 months. CT indicates computed tomography.

Our study was a single-center, open-label, randomized trial with a crossover design. Eligible patients were randomized to 1 of 2 groups (Figure 1): observation for 6 months followed by chemotherapy for 6 months (observation-first group) or chemotherapy for 6 months followed by observation for 6 months (chemotherapy-first group). Randomization was performed through the Computer Randomization Enrollment automated telephone randomization system.^34^ All patients were to be treated with a fluoropyrimidine-based regimen; the specific regimen was selected by the treating physician. Notably, this study was not blinded. With the data and safety monitoring board’s permission, the trial was administratively closed after 8 years.

### End Points and Assessments

The primary end point was the difference in tumor growth (percentage change), using the mpRECIST method, between the chemotherapy and observation periods. The mpRECIST was developed for this trial specifically to address the limitations of standard RECIST in appendix cancer. mpRECIST measures 5 lesions (mucinous and cystic lesions are allowed) in the peritoneal cavity in contrast to the maximum of 2 lesions, per standard RECIST. A computed tomography scan of the abdomen and pelvis was performed at baseline and every 3 months as standard of care. Tumor markers (carcinoembryonic antigen [CEA], cancer antigen 125 [CA125], and CA19-9) were measured in peripheral blood collected at baseline and 3, 6, 9, and 12 months. All patients with available 3-, 6-, 9-, or 12-month data were combined to compare percentage change in each marker level between the observation and treatment periods. Additional secondary efficacy end points were the objective response rate, the rate of bowel complications (defined as bowel obstruction requiring hospitalization or bowel perforation), and differences in OS between early and delayed chemotherapy approaches. Safety monitoring was conducted for the composite safety end point of death or bowel complication.

### Patient-Reported Outcomes

An additional secondary end point was difference in quality of life (QOL) between the treatment and observation periods. QOL was determined using 3 different questionnaires: the European Organization for Research and Treatment of Cancer Core Quality of Life Questionnaire (EORTC QLQ-C30); the ovarian cancer-specific EORTC QOL questionnaire (EORTC QLQ-OV28), due to the considerable similarity in symptoms of peritoneal disseminated disease from ovarian cancer, and the anxiety-specific Speilberger State/Trait Anxiety Inventory State (STAI) scale.^35,36^ Patients completed the 3 questionnaires at baseline and every 3 months. The EORTC QLQ-C30 ranges from 1 to 100, with higher overall score indicating better level of functioning overall, while higher scores on the symptom and single-item scales indicate a higher level of symptoms. The EORTC QLQ-OV28 ranges from 1 to 100, with higher score indicating greater symptom severity. The STAI ranges from 20 to 80, with higher score indicating higher anxiety.

### Statistical Analysis

To estimate effect size, 2 readers (K.P.R. and M.J.O.) retrospectively calculated mpRECIST in 5 patients with low-grade mucinous AA. The mean change in tumor size over a 6-month time period in patients receiving treatments was a 1.6% increase; in those without treatment, the increase was 9%. Based on these preliminary data, a 7.4% (95% CI, 3.0%-11.7%) effect size was observed. The SD of residuals was 3.5% for the random effects introduced by the 2 readers. Considering both the variation introduced by different readers and variation of the treatment effects, the combined SD of differences was 4.1%. Based on these preliminary data, we deemed a difference of at least 5% in mpRECIST-determined tumor size change to be clinically meaningful. Assuming a crossover analysis of variance square root of mean square error of 4.0% and a 1-sided α = .05, it was estimated that 24 patients would provide 80% power to detect a 5% difference; enrollment of up to 30 patients was planned to have complete 6- and 12-month tumor measures for 24 patients.

Crossover analyses were performed according to Senn methods.^37^ First, a formal test of interaction and visual inspection for period effect were performed to determine whether the treatment groups (observation first and chemotherapy first) could be combined for the test of observation vs treatment. Subsequently, paired *t* tests were used to compare tumor growth after 6 months of observation vs tumor growth after 6 months of treatment. Patients who did not complete the entire 12-month study period were not included in the primary efficacy analysis. A secondary efficacy analysis, including all patients who had any 6-month information, was performed using a generalized linear model accounting for the repeated measures for patients with both measures.

A safety monitoring rule was in place to stop the trial early if a Fisher exact test ever detected a difference between the treatment and observation period in this composite measure that was ever significant at the α = .05 level. OS was estimated in each group and graphed by Kaplan-Meier methods. Comparison between the treatment first and observation first groups was performed with a log-rank test. Kaplan-Meier curves were implemented in Stata statistical software version 16 (StataCorp). All other analyses were performed in SAS statistical software version 9.4 (SAS Institute). Data were analyzed from November 2021 to May 2022.

## Results

### Patient Characteristics and Disposition

Between December 2013 and January 2021, a total of 24 patients were enrolled in the study, with median (range) age of 63 (38-82) years and an equal proportion of men and women (eg, 12 men [50%]); all patients had ECOG performance status of 0 or 1. Eleven patients were randomized to chemotherapy first, and 13 patients were randomized to observation first. Most patients (20 patients [83%]) had well-differentiated tumors, and 4 patients (17%) had well-to-moderately differentiated tumors. Pathological diagnosis was confirmed by a pathologist with specific expertise in appendiceal cancer, and graded using a 3-tiered system evaluating tumor cellularity, destructive invasion, presence of signet ring cells, as well as complexity of tumor architecture^38^ (eTable 1 in Supplement 1). Sixteen patients had tumor alteration testing performed as part of standard-of-care treatment. The most frequently altered genes were *KRAS* (11 patients [69%]) and *GNAS* (8 patients [57%]) (eTable 2 in Supplement 1).^2,8^ Notably, nearly all of the patients had prior CRS (22 patients [92%]) (eTable 3 in Supplement 1). There was a wide range in the time from diagnosis to randomization, with 3 patients (13%) randomized within 6 months of diagnosis and 6 patients (25%) randomized more than 5 years after initial diagnosis. After randomization, the chemotherapy-first and observation-first groups were balanced with respect to these categories (eTable 4 in Supplement 1). Due to slow accrual, after 8 years of recruitment, the trial was stopped with 24 of the planned 30 patients enrolled. Three patients withdrew consent prior to completing the first 6-month period and were excluded from the primary end point analysis (all 3 patients were unable to maintain the follow-up schedule). Two patients in the observation-first group completed the observation period but then declined chemotherapy treatment, and 1 patient declined traveling to MDACC for follow-up (Figure 1). Most patients (15 patients [63%]) were treated with either fluorouracil or capecitabine as single agent ; bevacizumab was added for 3 patients (13%), 1 patient was treated with leucovorin calcium (folinic acid), fluorouracil, and oxaliplatin, and 2 patients (8%) were treated with folinic acid, fluorouracil, and irinotecan hydrochloride.

### Efficacy

**Figure 2.**
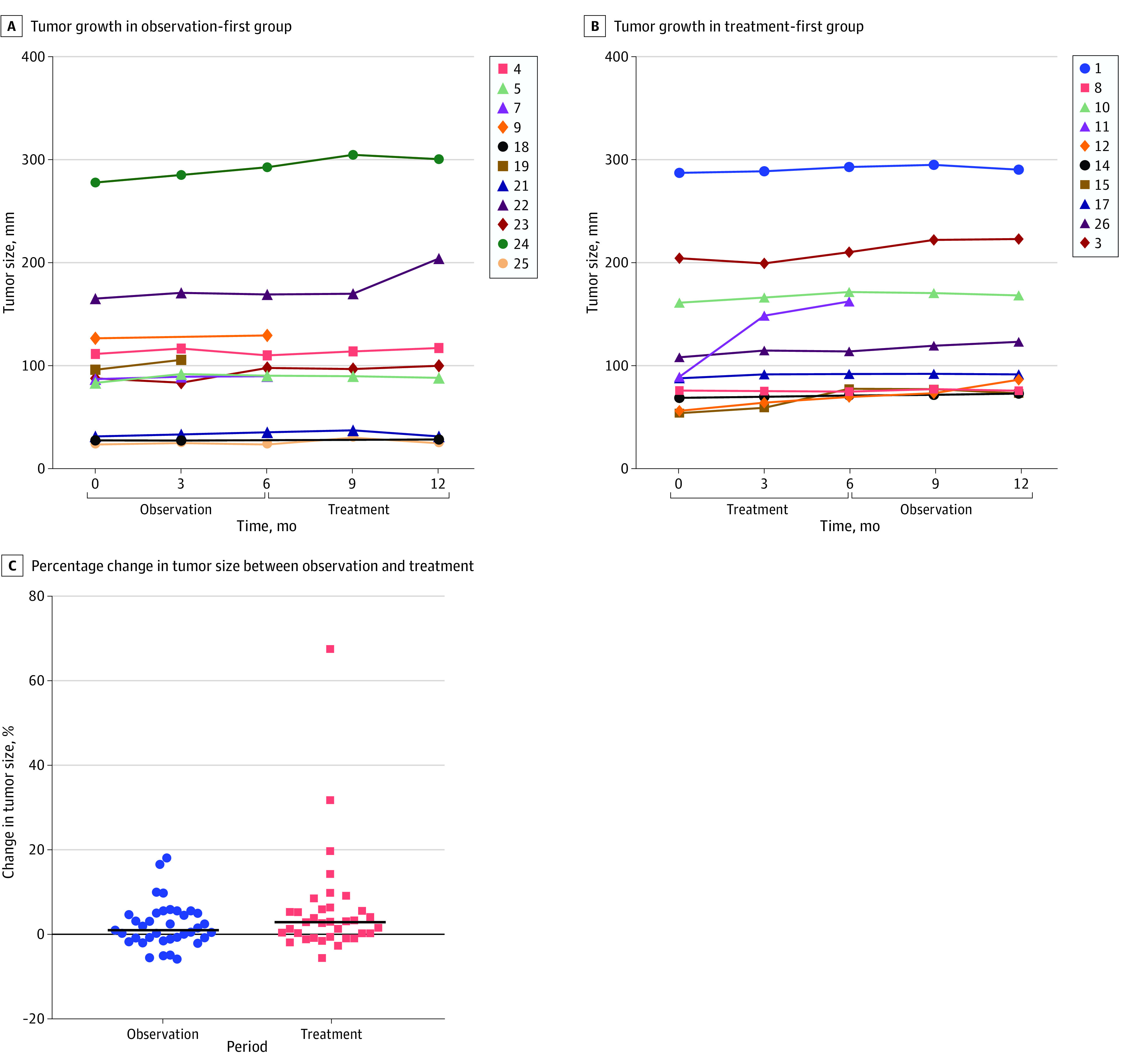
Tumor Growth and Percentage Change in Tumor Size Between Observation and Treatment Groups Measured using modified peritoneal Response Evaluation Criteria in Solid Tumors, a novel quantitative measuring system designed for mucinous peritoneal disease, which measures up to 5 areas of mucinous disease in the abdominal cavity. Numbers indicate patient IDs.

Fifteen patients completed the full 12-month study period and were available to evaluate the primary end point of difference in tumor growth between treatment and observations periods; there was not a significant difference (8.4%; 95% CI, 1.5%-15.3% vs 4.0% 95% CI, −0.1% to 8.0%; *P* = .26) (eTable 5 in Supplement 1). The interaction between timing and treatment was not statistically significant in the crossover analysis and minimal on visual inspection (eFigure 2 in Supplement 1). A simple paired analysis of the percentage change in tumor volume during treatment vs observation was conducted for all 18 patients with measurements in both conditions. Similarly, rate of tumor growth was not significantly different between treatment and observation periods (13.1%; 95% CI, 1.3% to 25.0%; vs 4.4%; 95% CI, 0.8% to 8.1%; *P* = .37) (Figure 2). In total, 18 patients received chemotherapy during the study period and none achieved an objective response, 14 patients (77.8%) had stable disease during the entire year of follow up, 4 patients (12.2%) had progression during the study (eFigure 5 in Supplement 1).

**Figure 3.**
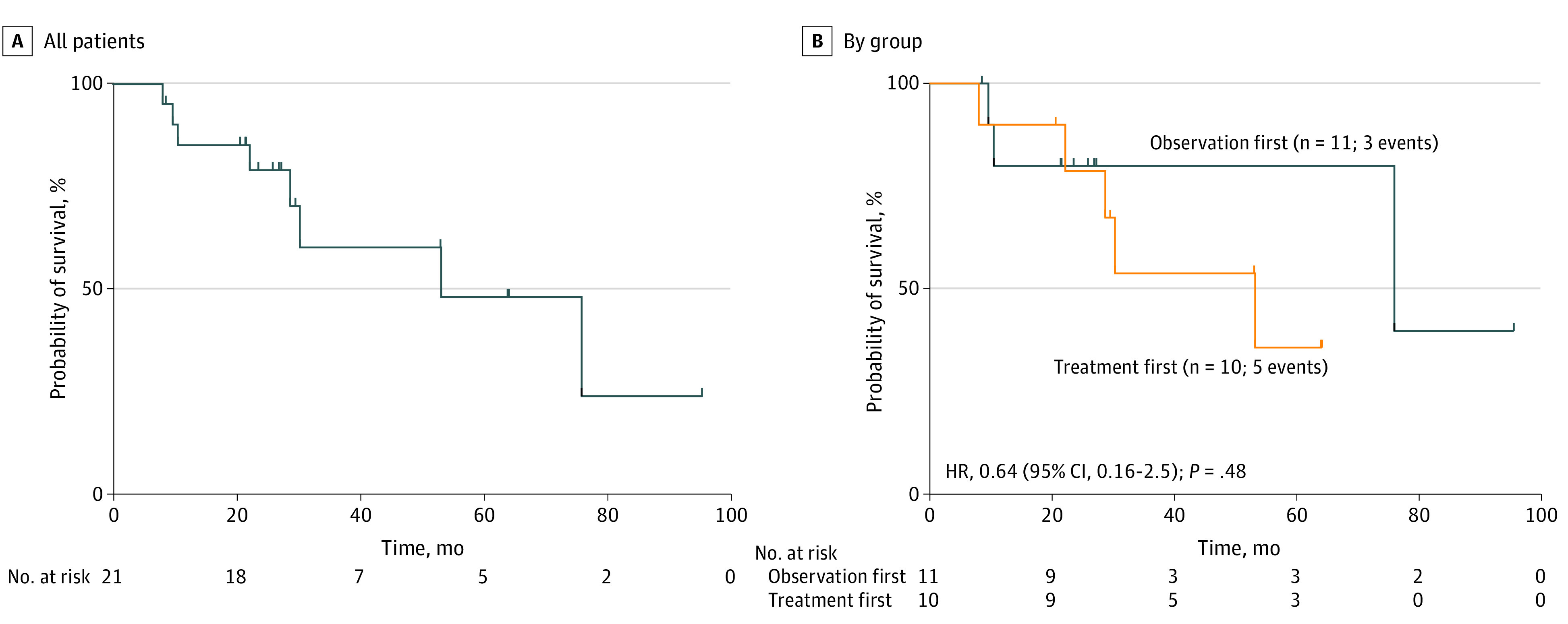
Kaplan-Meier Curves Showing Overall Survival of All Patients and Between Groups Crosses indicate censoring.

**Figure 4.**
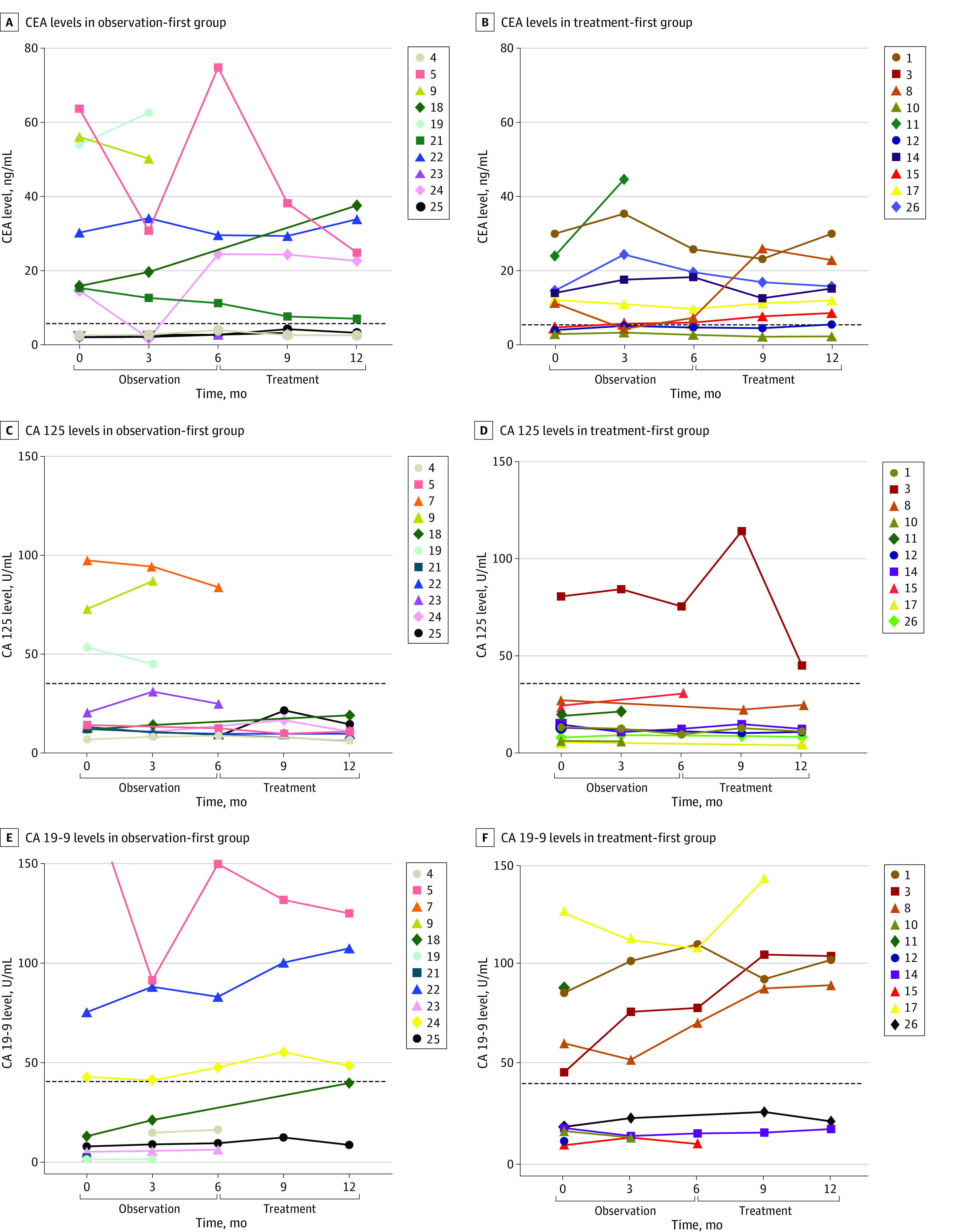
Spider Plots Showing Tumor Markers Level Over Time CA indicates cancer antigen; CEA, carcinoembryonic antigen.

Median (range) OS for the entire cohort was 53.2 (8.1 to 95.5) months, and there was no significant difference in OS between the observation-first group (76.0 [8.6 to 95.5] months) and the treatment-first group (53.2 [8.1 to 64.1] months; hazard ratio [HR], 0.64; 95% CI, 0.16 to 2.6; *P* = .48). The median (range) duration of follow up after study completion at the time of data cutoff was 27 (8 to 95) months, and only 3 patients (14.3%) received further systemic treatment after the trial (Figure 3; eFigure 4 in Supplement 1). There was not a significant difference between observation and treatment periods for the percentage change in any of the tumor markers evaluated (ie, CEA, CA125, and CA19-9) (eFigure 3 in Supplement 1). Notably the 2 patients with greatest elevation in CEA and CA125 (Figure 4) had markedly worse outcome, with an OS of 22 months for CEA and 10 months for CA 125.

### Safety

The composite safety outcome measure was similar between treatment and observation and between groups (eTable 6 in Supplement 1). Three patients who came off trial, 1 for declining chemotherapy and 2 for progression, died during the 12-month trial window. No patients had a bowel perforation during treatment or observation. Four patients were hospitalized for bowel obstruction, 2 each from the treatment-first and observation-first groups.

### Patient-Reported Outcomes

**Figure 5.**
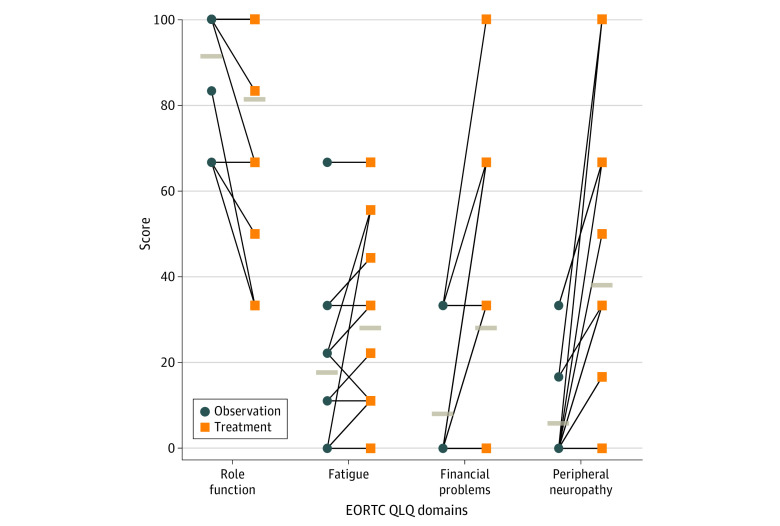
Quality of Life Questionnaire Individual Scores Between Observation and Treatment Periods Tan bars indicate mean score. EORTC QLQ indicates European Organization for Research and Treatment of Cancer Core Quality of Life Questionnaire, which ranges from 1 to 100, with higher subscale scores indicating worse levels of functioning.

Fifteen patients completed the patient-reported outcome questionnaires at both 6 and 12 months and were available for paired analyses. EORTC QLQ-C30 role function score, fatigue score, and financial difficulties scores were significantly increased during treatment relative to observation indicating worse quality of life while receiving chemotherapy (Figure 5); mean (SD) scores following observation vs following treatment were 92.2 (13.9) vs 82.2 (25.6) for role function (*P* = .03), 18.5 (18.6) vs 28.9 (21.3) for fatigue (*P* = .02), and 8.9 (15.2) vs 29.9 (33.0) for financial difficulties (*P* = .01) (eTable 7 in Supplement 1). EORTC QLQ-OV28 scores for peripheral neuropathy (mean [SD], 6.67 [12.28] vs 38.89 [34.88]; *P* = .001) and chemotherapy side effects (mean [SD], 16.45 [17.04] vs 23.11 [17.25]; *P* = .008) were significantly higher during treatment than during observation (eTable 8 in Supplement 1). There was no significant difference in STAI scores between observation and treatment periods (eTable 9 in Supplement 1).

## Discussion

This randomized crossover trial found that fluoropyrimidine-based chemotherapy was not effective for patients with low-grade mucinous AA, as there was not a single objective response and not a significant difference in tumor growth while during chemotherapy vs observation. Due both to the rarity and heterogeneity of AA, it has been difficult to objectively determine whether systemic chemotherapy is effective in the treatment of this disease. This study represents the first prospective, randomized trial for low-grade mucinous AA to answer this pivotal question, to our knowledge. Moreover, chemotherapy significantly decreased QOL during the chemotherapy period compared with the observation period. Similarly, delaying the start of chemotherapy with a 6-month observation period did not reduce OS nor increase rate of bowel perforation or obstruction.

The results from this prospective, randomized trial are consistent with multiple prior retrospective analyses suggesting chemotherapy is ineffective in low-grade AA.^14,15,17,18,28,39^ A study by Shaib et al^16^ found that patients with metastatic low-grade appendiceal mucinous neoplasms who did not receive systemic chemotherapy had longer median OS than those who did.^16^ Similarly, a retrospective study^15^ that included 1919 patients with metastatic low-grade mucinous AA from the National Cancer Database from 1985 to 2006 found chemotherapy was not associated with improved survival ).^15^ A 2019 analysis of National Cancer Database data from 2004 to 2015, including 639 patients with metastatic low-grade mucinous AA, confirmed this lack of survival benefit.^18^ With respect to perioperative systemic chemotherapy, in a retrospective analysis of 104 patients with PMP of mixed grades who underwent CRS or HIPEC in a multivariate analysis, including grade, preoperative chemotherapy was associated with worse survival.^14^ Similarly, a retrospective study of perioperative chemotherapy in 284 patients with mucinous PMP patients found that for the 22 patients with low-grade disease treated with systemic chemotherapy, there was no difference in either OS or progression-free survival compared with a matched cohort without chemotherapy.^17^

To our knowledge there are no reports of objective response from cytotoxic systemic chemotherapy specifically in low-grade AA. It is important to note that this is in contrast to high-grade AA, which is known to be responsive to cytotoxic chemotherapy on the basis of many prior reports.^28,40^ There is only 1 other published prospective trial of systemic chemotherapy in unresectable PMP, to our knowledge: a single-group phase II study of mitomycin C and capecitabine, which found tumor reduction in 15% of patients, stable disease in 45% of patients and progression in 28% of patients.^41^ However, this study included a mixed population of tumors, with 32% of higher-grade mucinous AA classified as mucinous carcinoma peritonei and 68% of low-grade mucinous AA classified as disseminated peritoneal adenomucinous.^19,42^ This study did not breakdown the response by histology, so it is unclear if any of the responding patients had low-grade tumors. Although several retrospective studies that combined both high- and low-grade AA have reported an aggregate benefit to chemotherapy,^5,41,43^ the results of this prospective study and growing recognition of the molecular and clinical differences between high- and low-grade AA^2,8^ argue that these 2 distinct subtypes should not be grouped together.^44^

### Limitations

This study has some limitations. The trial was initially planned for enrollment of 30 patients to have complete data for 24 patients, providing 80% power for the primary end point. However, accrual in this rare disease was slow, with only 24 patients enrolled after 8 years (2013 to 2021). When designed in 2012, there was concern that delaying start of treatment would harm patients, thus the only prespecified interim analyses concerned increased death or severe complication in the observation first group. Although not prespecified, since the trial was not blinded, interim efficacy analysis was performed after 8 years; given complete lack of response to chemotherapy and no difference in tumor growth between observation and treatment periods, the trial was closed, as it was felt unethical to continuing treating patients with low-grade AA with fluorouracil-based chemotherapy. This trial was under the institutional data and safety monitoring board oversight for annual review of safety and efficacy. With the data and safety monitoring board’s permission, the trial was administratively closed. All of the patients in the study were enrolled at a tertiary referral cancer center, which may not be representative of patients in a community oncology practice. Despite these limitations, our study represents the first prospective, randomized trial for low-grade AA, to our knowledge.

## Conclusions

The findings of this randomized crossover trial, taking into consideration the lack of benefit from fluoropyrimidine-based chemotherapy seen in this trial and prior retrospective studies with similar conclusion, suggest that fluoropyrimidine-based chemotherapy should not be considered a standard-of-care treatment for patients with low-grade AA who are not candidates for CRS. Clinical trials investigating novel therapeutics should be considered for these patients. These prospective clinical data highlight the differences between AA and CRC and demonstrate the need for the development of appendiceal cancer–specific guidelines as well as more preclinical and clinical investigation for this disease.^45,46,47,48,49,50,51,52,53,54,55^ An additional important observation for the clinical management of low-grade AA is that, given natural slow growth of these tumors and the difficulty of imaging peritoneal carcinomatosis, stable disease, as assessed by computed tomography or magnetic resonance imaging, cannot be interpreted as clinical benefit in low-grade AA, as it is in most other solid tumors. In summary, the data from this prospective, randomized crossover trial suggest that patients with low-grade mucinous AA did not benefit from systemic fluoropyrimidine-based chemotherapy.

## Notes

Supplement 1. **eFigure 1.** Low-Grade Mucinous Appendiceal Adenocarcinoma **eFigure 2.** Interaction Between Time and Treatment **eFigure 3.** Waterfall Plots Showing Tumor Markers Percentage Change Between Observation and Treatment Periods **eFigure 4.** Best Overall Response After Treatment Period and Treatment History Over Time for Both Groups **eTable 1.** Patients Histopathology and Grade **eTable 2.** Tumor Molecular Profile **eTable 3.** Prior Cytoreductive Surgery History of All Patients **eTable 4.** Patients Characteristics by Randomized Treatment Group **eTable 5.** Tumor Measure Availability and Percentage Change for Evaluable Patients **eTable 6.** Numbers of Patients With Monitored Adverse Events, Counted Once Per Treatment Period **eTable 7.** Paired *t* Test for QLQ C-30 **eTable 8.** Paired *t* Test for QLQ OV-28 **eTable 9.** Paired *t* Test for STAI

Supplement 2.
Trial Protocol and Statistical Analysis Plan

Supplement 3.
Data Sharing Statement
