# Supplementary material for: Efficacy of Systemic Chemotherapy in Patients With Low-grade Mucinous Appendiceal Adenocarcinoma: A Randomized Crossover Trial
Source: JAMA Netw Open. 2023 Jun 1;6(6):e2316161. doi: 10.1001/jamanetworkopen.2023.16161 (PMC10236240; doi:10.1001/jamanetworkopen.2023.16161)
Supplement: Supplement 1. — eFigure 1. Low-Grade Mucinous Appendiceal Adenocarcinoma eFigure 2. Interaction Between Time and Treatment eFigure 3. Waterfall Plots Showing Tumor Markers Percentage Change Between Observation and Treatment Periods eFigure 4. Best Overall Response After Treatment Period and Treatment History Over Time for Both Groups eTable 1. Patients Histopathology and Grade eTable 2. Tumor Molecular Profile eTable 3. Prior Cytoreductive Surgery History of All Patients eTable 4. Patients Characteristics by Randomized Treatment Group eTable 5. Tumor Measure Availability and Percentage Change for Evaluable Patients eTable 6. Numbers of Patients With Monitored Adverse Events, Counted Once Per Treatment Period eTable 7. Paired t Test for QLQ C-30 eTable 8. Paired t Test for QLQ OV-28 eTable 9. Paired t Test for STAI [file jamanetwopen-e2316161-s001.pdf]

Supplemental Online Content

Shen JP, Yousef AM, Zeineddine FA, et al. Efficacy of systemic chemotherapy in patients with low-grade mucinous appendiceal adenocarcinoma: a randomized crossover trial. *JAMA Netw Open*. 2023;6(6):e2316161. doi:10.1001/jamanetworkopen.2023.16161

eFigure 1. Low-Grade Mucinous Appendiceal Adenocarcinoma
eFigure 2. Interaction Between Time and Treatment
eFigure 3. Waterfall Plots Showing Tumor Markers Percentage Change Between Observation and Treatment Periods
eFigure 4. Best Overall Response After Treatment Period and Treatment History Over Time for Both Groups
eTable 1. Patients Histopathology and Grade
eTable 2. Tumor Molecular Profile
eTable 3. Prior Cytoreductive Surgery History of All Patients
eTable 4. Patients Characteristics by Randomized Treatment Group
eTable 5. Tumor Measure Availability and Percentage Change for Evaluable Patients
eTable 6. Numbers of Patients With Monitored Adverse Events, Counted Once Per Treatment Period
eTable 7. Paired *t* Test for QLQ C-30
eTable 8. Paired *t* Test for QLQ OV-28
eTable 9. Paired *t* Test for STAI

This supplemental material has been provided by the authors to give readers additional information about their work.

© 2023 Shen JP et al. *JAMA Network Open.*

eFigure 1. Low-Grade Mucinous Appendiceal Adenocarcinoma

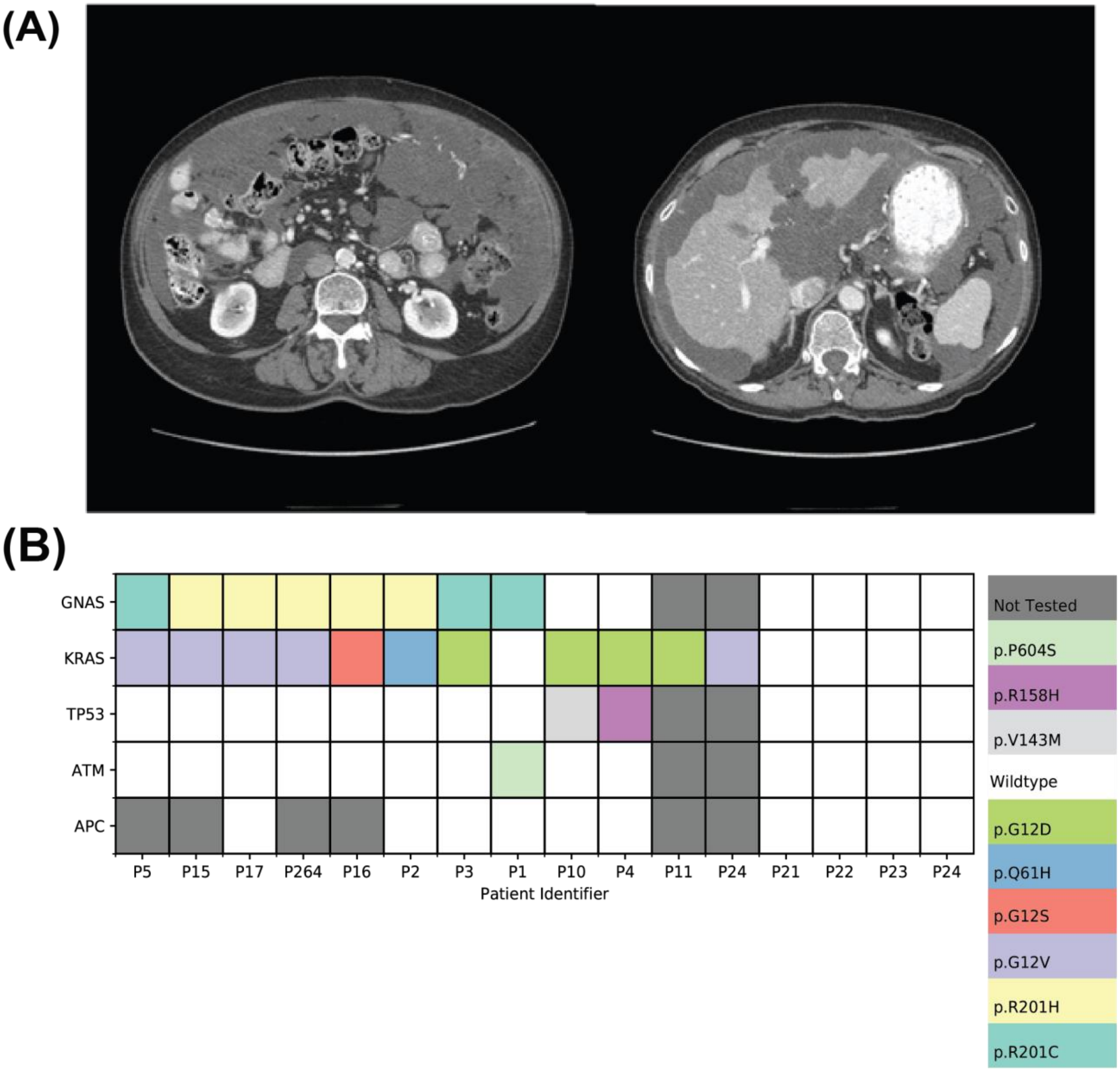

(A) CT scans highlighting diffuse mucinous nature of this tumor type. (B) 16 patients on study has standard-of-care mutation testing, dark grey indicates gene was not tested for that patient, white indicates gene was tested and found to be wildtype.

© 2023 Shen JP et al. *JAMA Network Open.*

eFigure 2. Interaction Between Time and Treatment

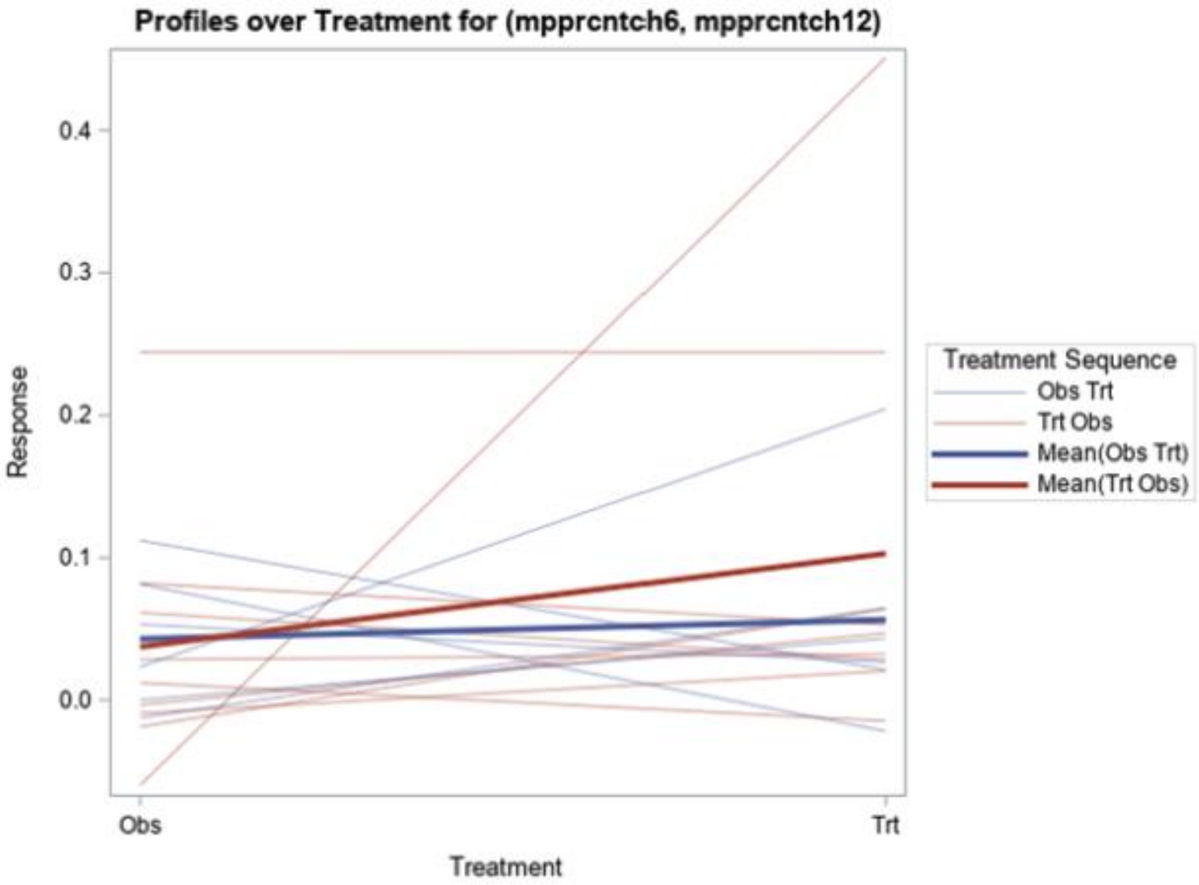

© 2023 Shen JP et al. *JAMA Network Open.*

eFigure 3. Waterfall Plots Showing Tumor Markers Percentage Change Between Observation and Treatment Periods

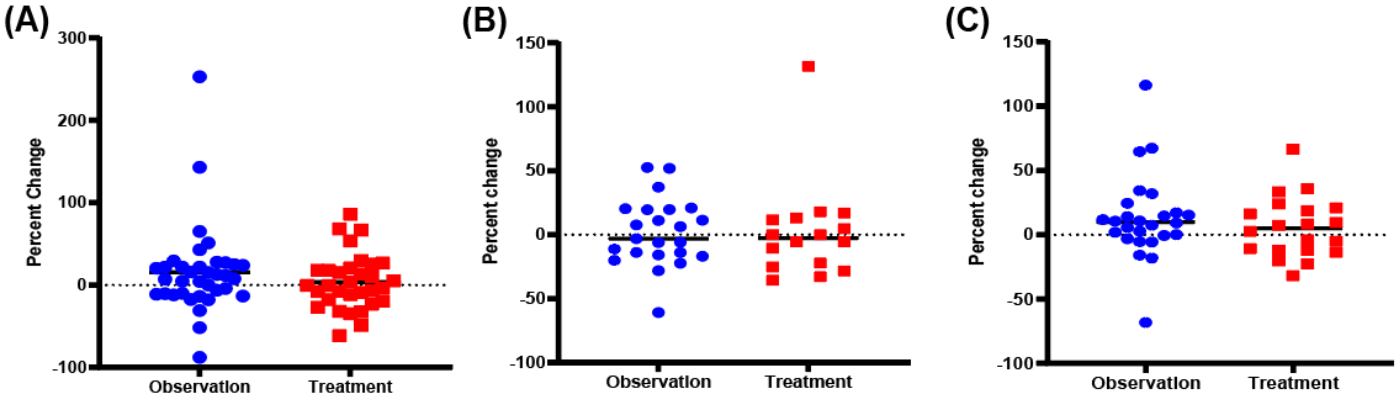

(A) CEA percent change in observation on the left and treatment on the right. (B) CA 125 percent change in observation on the left and treatment on the right. (C) CA19-9 percent change in observation on the left and treatment on the right.

© 2023 Shen JP et al. *JAMA Network Open.*

eFigure 4. Best Overall Response After Treatment Period and Treatment History Over Time for Both Groups

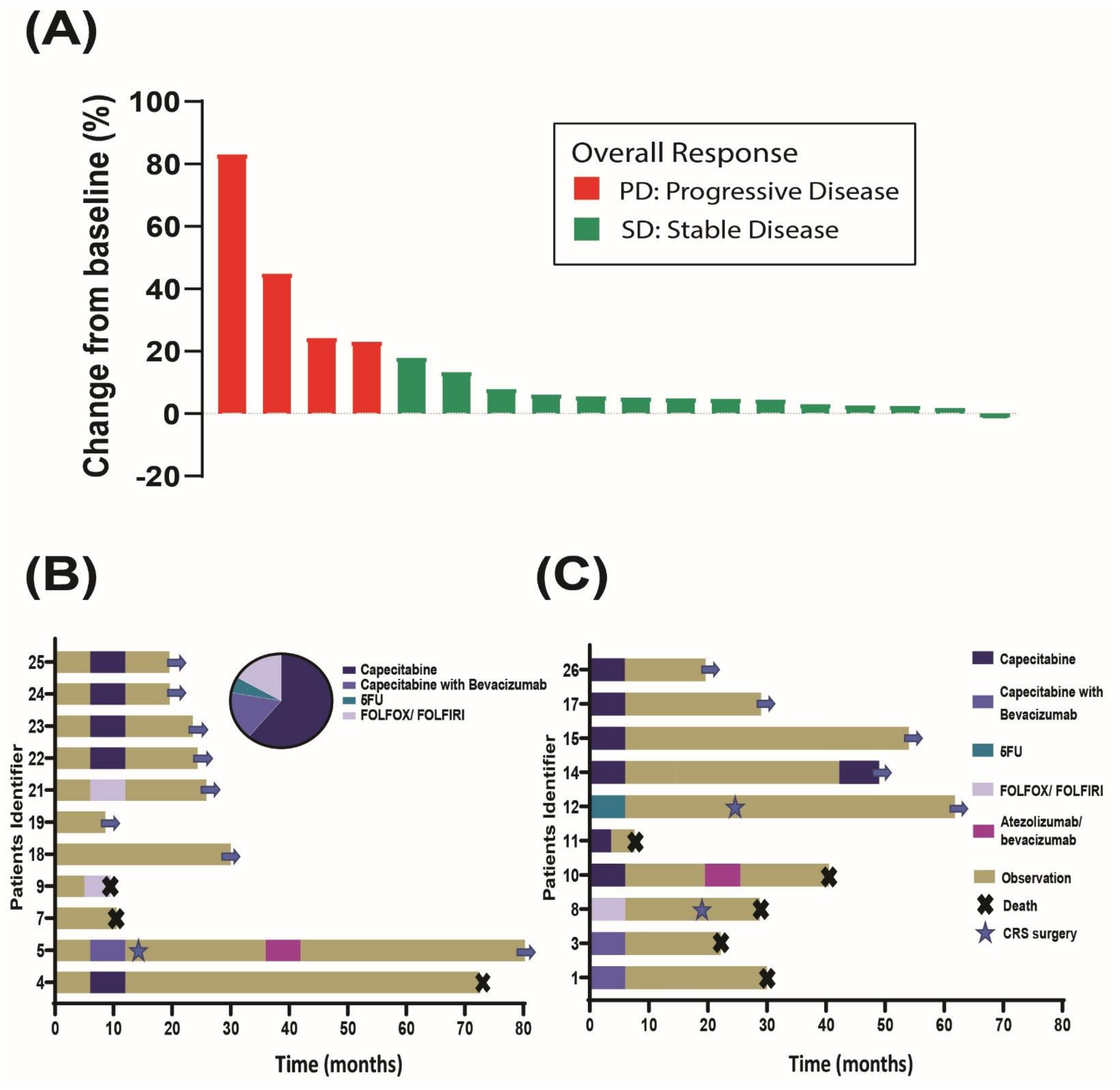

(A) Waterfall plot showing best overall response after treatment period. (B) Observation first arm treatment history overtime. (C) Treatment first arm treatment history overtime. The pie chart shows the chemotherapy distribution patients received during the trial period.

© 2023 Shen JP et al. *JAMA Network Open.*

eTable 1. Patients Histopathology and Grade

Patient Identifier	Histology	Grade
1	MUCINOUS ADENOCARCINOMA	WELL-TO-MODERATELY DIFFERENTIATED
2	MUCINOUS ADENOCARCINOMA	WELL-DIFFERENTIATED
3	MUCINOUS ADENOCARCINOMA	WELL-DIFFERENTIATED
4	MUCINOUS ADENOCARCINOMA	WELL-TO-MODERATELY DIFFERENTIATED
5	MUCINOUS ADENOCARCINOMA	WELL-DIFFERENTIATED
6	MUCINOUS ADENOCARCINOMA	WELL-DIFFERENTIATED
7	MUCINOUS ADENOCARCINOMA	WELL-DIFFERENTIATED
8	MUCINOUS ADENOCARCINOMA	WELL-DIFFERENTIATED
9	MUCINOUS ADENOCARCINOMA	WELL-DIFFERENTIATED
10	MUCINOUS ADENOCARCINOMA	WELL-DIFFERENTIATED
11	MUCINOUS ADENOCARCINOMA	WELL-DIFFERENTIATED
12	MUCINOUS ADENOCARCINOMA	WELL-DIFFERENTIATED
14	MUCINOUS ADENOCARCINOMA	WELL-DIFFERENTIATED
15	MUCINOUS ADENOCARCINOMA	WELL-DIFFERENTIATED
16	MUCINOUS ADENOCARCINOMA	WELL-DIFFERENTIATED
17	MUCINOUS ADENOCARCINOMA	WELL-DIFFERENTIATED
18	MUCINOUS ADENOCARCINOMA	WELL-TO-MODERATELY DIFFERENTIATED
19	MUCINOUS ADENOCARCINOMA	WELL-DIFFERENTIATED
21	MUCINOUS ADENOCARCINOMA	WELL-DIFFERENTIATED
22	MUCINOUS ADENOCARCINOMA	WELL-DIFFERENTIATED
23	MUCINOUS ADENOCARCINOMA	WELL-DIFFERENTIATED
24	MUCINOUS ADENOCARCINOMA	WELL-DIFFERENTIATED
25	MUCINOUS ADENOCARCINOMA	WELL-DIFFERENTIATED
26	MUCINOUS ADENOCARCINOMA	WELL-TO-MODERATELY DIFFERENTIATED

© 2023 Shen JP et al. *JAMA Network Open.*

eTable 2. Tumor Molecular Profile

Patient Identifier	Allocation	Genes					
		GNAS	KRAS	JAK3	TP53	ATM	APC
4	Observation First	Wildtype	**p.G12D**	Wildtype	**p.R158H**	wildtype	wildtype
5	Observation First	**p.R201C**	**p.G12V**	wildtype	wildtype	wildtype	N/A
16	Observation First	**p.R201H**	**p.G12S**	wildtype	wildtype	wildtype	N/A
18	Observation First	**p.R201H**	**p.G12V**	wildtype	wildtype	wildtype	N/A
21	Observation First	wildtype	wildtype	wildtype	wildtype	wildtype	wildtype
22	Observation First	wildtype	wildtype	**p.V617F**	wildtype	wildtype	wildtype
23	Observation First	wildtype	wildtype	wildtype	wildtype	wildtype	wildtype
24	Observation First	N/A	**p.G12V**	N/A	N/A	N/A	N/A
1	Treatment First	**p.R201C**	wildtype	wildtype	wildtype	**p.P604S**	wildtype
2	Treatment First	**p.R201H**	**p.Q61H**	wildtype	wildtype	wildtype	wildtype
3	Treatment First	**p.R201C**	**p.G12D**	wildtype	wildtype	wildtype	wildtype
10	Treatment First	Wildtype	**p.G12D**	wildtype	**p.V143M**	wildtype	wildtype
11	Treatment First	N/A	**p.G12D**	N/A	N/A	N/A	N/A
14	Treatment First	wildtype	wildtype	wildtype	wildtype	wildtype	wildtype
15	Treatment First	**p.R201H**	**p.G12V**	wildtype	wildtype	wildtype	N/A
26	Treatment First	**p.R201H**	**p.G12V**	wildtype	wildtype	wildtype	wildtype

© 2023 Shen JP et al. *JAMA Network Open.*

eTable 3. Prior Cytoreductive Surgery History of All Patients

Accession No	Prior CRS before Trial	1st CRS Date	CCR Score	PCI score	2nd CRS Date	CCR Score	PCI score
1	Yes	2013		30			
2	Yes	2005			2013		32
3	Yes	2013					
4	Yes	2012					
5	Yes	2013	0	20			
6	Yes	2011					
7	No						
8	Yes	2014	1	24			
9	Yes	2015	2	35			
10	Yes	2012	1	30			
11	Yes	2015	1	25			
12	Yes	2012	1	27			
14	Yes	2010	0	14	2015	0	24
15	Yes	2014	1	20			
16	Yes	2017	1	25			
17	Yes	2019		33			
18	Yes	2018	1	24			
19	No						
21	Yes	2016	1	17	2018	1	21
22	Yes	2019					
23	Yes	2019	1	21			
24	Yes	2010	0	25	2014	1	21
25	Yes	2014	1	15	2017	0	
26	Yes	2013	2	32			

CRS = Cytoreductive Surgery
CCR = Completeness of cytoreduction
PCI = Peritoneal Carcinomatosis Index

© 2023 Shen JP et al. *JAMA Network Open.*

eTable 4. Patients Characteristics by Randomized Treatment Group

Patient Characteristics		All<br>N (%)	Treatment First<br>N (%)	Observation First<br>N (%)
All		24 (100%)	11 (100%)	13 (100%)
Age - median (min, max)	N=24	62.8 (38.0, 81.8)	62.8 (44.8, 79.3)	60.9 (38.0, 81.8)
Race/ethnicity				
	Black or African	6 (25%)	2 (18%)	4 (31%)
	Hispanic	2 (8%)	1 (9%)	1 (8%)
	White or Caucasian	14 (58%)	7 (64%)	7 (54%)
	Other	2 (8%)	1 (9%)	1 (8%)
Sex				
	Female	12 (50%)	6 (55%)	6 (46%)
	Male	12 (50%)	5 (45%)	7 (54%)
ECOG				
	0	15 (63%)	8 (73%)	7 (54%)
	1	9 (38%)	3 (27%)	6 (46%)
Histology grade				
	Well to Moderate differentiated	4 (17%)	2 (18%)	2 (15%)
	Well differentiated	20 (83%)	9 (82%)	11 (85%)
Planned treatment				
	.	4 (17%)	0 (0%)	4 (31%)
	5FU with Bevacizumab	1 (4%)	0 (0%)	1 (8%)
	5FU without Bevacizumab	1 (4%)	1 (9%)	0 (0%)
	CAPOX with Bevacizumab	2 (8%)	1 (9%)	1 (8%)
	Capecitabine with Bevacizumab	2 (8%)	2 (18%)	0 (0%)
	Capecitabine without Bevacizumab	11 (46%)	6 (55%)	5 (38%)
	FOLFIRI without Bevacizumab	2 (8%)	0 (0%)	2 (15%)
	FOLFOX without Bevacizumab	1 (4%)	1 (9%)	0 (0%)
Observation period complete				
	No	6 (25%)	2 (18%)	4 (31%)
	Yes	18 (75%)	9 (82%)	9 (69%)
Treatment period complete				
	No	8 (33%)	2 (18%)	6 (46%)
	Yes	16 (67%)	9 (82%)	7 (54%)
Prior CRS		22 (92%)	11 (100%)	11 (85%)
Time from diagnosis to randomization (months)				
	0 to 6	3 (13%)	1 (9%)	2 (15%)
	7 to 23	9 (37%)	3 (27%)	6 (47%)
	24 to 60	6 (25%)	3 (27%)	3 (23%)
	>60	6 (25%)	4 (37%)	2 (15%)
KRAS and GNAS status	N = 16			
	KRAS Mutated	11 (69%)	6 (75%)	5 (63%)
	GNAS Mutated	8 (50%)	5 (63%)	3 (38%)

5FU = 5 Fluorouracil; CRS = Cytoreductive Surgery; Race other = Asian/Unknown

© 2023 Shen JP et al. *JAMA Network Open.*

eTable 5. Tumor Measure Availability and Percentage Change for Evaluable Patients

	Observation First<br>**N**	Treatment First<br>**N**	All<br>**N**
Baseline N	13	11	24
6 Months N	8	10	18
12 Months N	6	9	15
**Results for patients of primary analysis N=15**			
Test for period effect (treatment x period interaction)			P=0.60
% Change at 6m on Observation	4.3 (-0.8, 9.4)	3.7 (-3.0, 10.5)	4.0 (-0.1, 8.0)
% Change at 6m on Treatment	5.6 (-2.5, 13.8)	10.3 (-1.2, 21.8)	8.4 (1.5, 15.3)
% Change at 12m all available	10.0 (2.4, 17.7)	14.5 (0.1, 28.8)	12.7 (4.4, 21.0)
Test for treatment effect (treatment vs. observation)			P=0.26
**Results for all available patients N=18**			
% Change at 6m on Observation	5.1 (1.0, 9.3)	3.7 (-3.0, 10.5)	4.4 (0.8, 8.1)
% Change at 6m on Treatment	5.6 (-2.5, 13.8)	17.6 (-1.8, 36.9)	13.1 (1.3, 25.0)
% Change at 12m all available	9.0 (2.3, 15.7)	14.5 (0.1, 28.8)	12.1 (4.2, 19.9)
Test for treatment effect (treatment vs. observation)			P=0.37

© 2023 Shen JP et al. *JAMA Network Open.*

eTable 6. Numbers of Patients With Monitored Adverse Events, Counted Once Per Treatment Period

Toxicity		Treatment 1st	Observation 1st	Total
**N Evaluable***		**10**	**11**	**21**
**Bowel Obstruction****	**Observation**	**1**	**1**	**2**
	**Treatment**	**2**	**1**	**3**
**Bowel Perforation**	**Observation**	**0**	**0**	**0**
	**Treatment**	**0**	**0**	**0**
**Deaths*****	**Observation**	**0**	**0**	**0**
	**Treatment**	**0**	**0**	**0**
**Any Events**	**Observation**	**1**	**1**	**2**
	**Treatment**	**2**	**1**	**3**
**Off Study Early Deaths******	**Observation**	**1**	**0**	**1**
	**Treatment**	**0**	**2**	**2**

*** To be evaluable for toxicity monitoring, the patient either needed to have had an event or completed 3 months on study with no toxic event.**

**** Obstruction counted only when it required hospitalization. The table numbers include one patient on the treatment first arm who had bowel obstruction hospitalizations twice each on both treatment and observation, for a total of 8 obstruction events among 4 patients.**

***** Deaths counted only while the patient was actively on study treatment or observation.**

******Additional patients died within the 12-month window, but after going off the trial.**

© 2023 Shen JP et al. *JAMA Network Open.*

eTable 7. Paired *t* Test for QLQ C-30

	P value	Mean of Observation	Mean of Treatment	Difference	SE of difference	t ratio	df	q value
Global health status/QoL	0.770	75	73.33	1.665	5.587	0.2981	14	0.82602
Physical Function	0.070	90.11	84	6.111	3.119	1.96	14	0.26615
Role Function	0.033	92.22	82.22	10	4.242	2.358	14	0.168984
Emotional Function	0.653	86.67	88.89	-2.223	4.844	0.459	14	0.82602
Cognitive Function	0.685	83.33	85.56	-2.223	5.362	0.4147	14	0.82602
Social Function	0.138	92.22	85.56	6.667	4.241	1.572	14	0.418982
Fatigue	0.025	18.52	28.89	-10.37	4.125	2.514	14	0.168984
Nausea / vomiting	0.510	10	6.667	3.333	4.933	0.6757	14	0.773128
Pain	0.818	14.44	15.56	-1.111	4.732	0.2347	14	0.82602
Dyspnoea	0.189	14.29	21.43	-7.143	5.157	1.385	13	0.47814
Insomnia	0.806	22.22	24.44	-2.221	8.889	0.2499	14	0.82602
Appetite loss	0.271	4.444	11.11	-6.667	5.819	1.146	14	0.515464
Constipation	0.272	11.9	19.05	-7.144	6.23	1.147	13	0.515464
Diarrhoea	0.510	15.56	22.22	-6.666	9.867	0.6756	14	0.773128
Financial problems	0.014	8.888	28.89	-20	7.127	2.806	14	0.168984

eTable 8. Paired *t* Test for QLQ OV-28

	P value	Mean of Observation	Mean of Treatment	Difference	SE of difference	t ratio	df	q value
Abdominal / GI	0.986	19.26	19.33	-0.07533	4.193	0.01797	14	0.865642
Peripheral neuropathy	0.001	6.667	38.89	-32.22	8.045	4.005	14	0.007895
Hormonal	1.000	14.44	14.44	0.0006667	6.3	0.000106	14	0.865642
Body image	0.334	15.56	11.11	4.443	4.444	0.9998	14	0.417319
Attitude to disease/ treatment	0.344	23.7	28.89	-5.185	5.297	0.9787	14	0.417319
Chemotherapy side effects	0.008	16.45	23.11	-6.667	2.158	3.09	14	0.024232
Other single items	0.081	5.185	13.15	-7.963	4.242	1.877	14	0.1646

eTable 9. Paired *t* Test for STAI

	P value	Mean of Observation	Mean of Treatment	Difference	SE of difference	t ratio	df
STAI	0.218	50.71	49.74	0.96	0.74	1.291	14
TAIY	0.068	52.57	51.22	1.35	0.68	1.979	14

© 2023 Shen JP et al. *JAMA Network Open.*
