# Supplementary material for: Efficacy of Systemic Chemotherapy in Patients With Low-grade Mucinous Appendiceal Adenocarcinoma: A Randomized Crossover Trial
Source: JAMA Netw Open. 2023 Jun 1;6(6):e2316161. doi: 10.1001/jamanetworkopen.2023.16161 (PMC10236240; doi:10.1001/jamanetworkopen.2023.16161)
Supplement: Supplement 2. — Trial Protocol and Statistical Analysis Plan [file jamanetwopen-e2316161-s002.pdf]

The University of Texas MD Anderson Cancer Center
Division of Cancer Medicine

Title: A Randomized Crossover Trial of Systemic Chemotherapy in Patients with Metastatic Well-differentiated or Moderately differentiated Mucinous Appendiceal Adenocarcinomas with Pseudomyxoma Peritonei.

Co-Principal Investigators

Michael J. Overman, MD
Assistant Professor, Department of Gastrointestinal Medical Oncology
The University of Texas MD Anderson Cancer Center
1515 Holcombe Blvd.
Unit # 426
Houston, TX 77030
Phone: 713-745-4317
Fax: 713-745-1163
moverman@mdanderson.org

Keith Fournier, MD
Assistant Professor, Surgical Oncology
The University of Texas MD Anderson Cancer Center
1515 Holcombe Blvd.
Unit # 1484
Houston, TX 77030
Phone: 713-792-8826
Fax: 713-794-1252
kffourni@mdanderson.org

Collaborators: Robert A. Wolff MD; Aurelio Matamoros MD; Tara Sagebiel MD; Paul Mansfield MD; Rebecca Slack, MS; Kanwal Raghav MD.

TABLE OF CONTENTS

1.0 Background

2.0 Objectives

3.0 Patient Eligibility

4.0 Study Design

5.0 Evaluation During Study

6.0 Criteria for Removal from the Study

7.0 Response Evaluation

8.0 Statistical Methods

9.0 Data and Protocol Management

10.0 Correlative Studies

11.0 References

APPENDICES

Appendix A – ECOG Performance Status

Appendix B – Survey Demographic Page

Appendix C - QLQ-C30, QLQ-OV28, and Speilberger State/Trait Anxiety Inventory State (STAI) Questionnaires

Appendix D - Mishel Uncertainty in Illness Scale (MUIS) Questionnaire

1 Background

1.1 Appendix Cancer: Overview

Appendiceal tumors are rare. The age-adjusted incidence of appendiceal malignancies is about 0.12 cases per 1,000,000 per year.[1] Tumors of the appendix are present in 0.9% of all appendectomy specimens and one-fourth to one-third of these are appendiceal epithelial neoplasms (AEN).[1,2]

The term AEN incorporates a spectrum of tumors ranging from benign appendiceal mucoceles to mucinous cystadenomas to well-differentiated adenocarcinomas to poorly differentiated signet ring cell adenocarcinomas (Figure 1). The appendiceal adenocarcinomas in turn can be either mucinous type (producing abundant mucin) or colonic/intestinal type.[1] This classification system is based on histological appearance and clinical behavior and is not incontrovertible.

The clinical course of AEN differs according to histological type and can vary from being relatively indolent to highly aggressive and dictates survival. The 5-yr disease specific survival of mucinous adenocarcinomas is about 44-58% in comparison to 20-27% for signet ring cell carcinoma.[1] The 5-year survival rate is also dependent on grade (68% for Grade 1 and 7% for Grade 3).[3] Data also indicates that patients with mucinous type have a better prognosis than those with colonic type appendiceal adenocarcinoma.[3]

Owing to their rarity, etiology, risk factors and prognostic factors for appendix cancers is poorly understood thus far. In clinical practice these tumors are generally perceived as an analog of colorectal cancer (CRC) and as a result management principles of CRC are applied to management of AEN. However, AEN have a markedly different biology and natural history than CRC.

1.2 Appendix Cancer: PMP

Pseudomyxoma peritonei (PMP) is a unique clinical entity, characterized by diffuse intraperitoneal collection of gelatinous material and mucinous implants on the peritoneal surfaces resulting from peritoneal dissemination of mucin-producing appendiceal cystadenomas and mucus-producing appendiceal adenocarcinoma. The progressive accumulation of copious amounts of mucinous fluid causes abdominal distention and eventually bowel obstruction and failure, which is fatal without treatment.[4]

The unique features of PMP include the following:

- These are slow growing tumors with a median survival in the range of 5-10 years.
- These are histologically characterized by accumulation of abundant extracellular mucin and not by accumulation of cancer cells (Figure 1)
- The pattern of metastatic spread is also unique and is usually confined to the peritoneal cavity. Hematogenous spread to liver or lungs or lymph node metastasis is extremely unusual.

Figure 1: Mucinous peritoneal disease in a patient with well differentiated adenocarcinoma with the clinical syndrome of PMP.

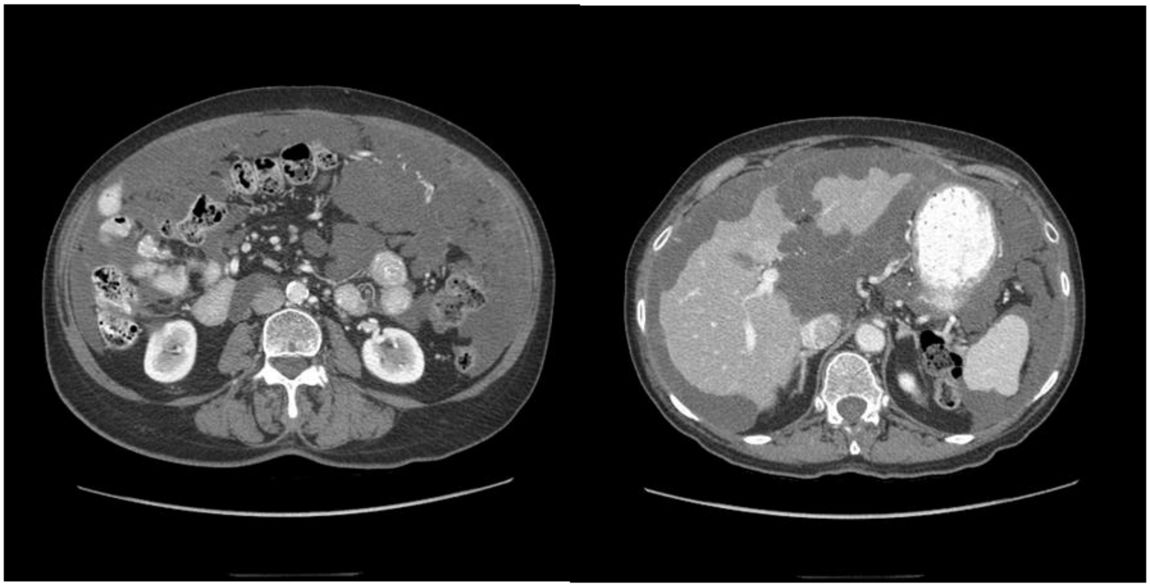

The classification of PMP is controversial and multiple overlapping systems exist.[5] There are some series that make a distinction between disseminated peritoneal adenomucinosis (DPAM) and peritoneal mucinous carcinomatosis (PMCA), both of which can present as pseudomyxoma peritonei.[6] DPAM are characterized by peritoneal lesions composed of abundant extracellular mucin containing scant simple to focally proliferative mucinous epithelium with little cytologic atypia or mitotic activity, with or without an associated appendiceal mucinous adenoma and PMCA are characterized by peritoneal lesions composed of more abundant mucinous epithelium with the architectural and cytologic features of carcinoma, with or without an associated primary mucinous adenocarcinoma.[6] This histological distinction does result in differences in age-adjusted 5-year survival rates of 84% for patients with DPAM, 37.6% for PMCA with intermediate features (PMCA-I) and 6.7% for patients with PMCA.[6,7] When combining both mucin-producing appendiceal cystadenomas (or DPAM) and mucinous well-differentiated appendiceal adenocarcinomas (or PMCA-I) the outcome for this group is indolent with 5-year overall survival of 78% with cytoreductive surgery.[7]
At MD Anderson the PMCA staging system is not utilized, and instead patients with PMCA are grade as well, moderate, or poorly differentiated. Well-differentiated mucinous adenocarcinomas are similar to the PMCA-I category and will represent the population of patients studied in this protocol.

1.3 PMP: Management

1.3.1 Cytoreductive Surgery

Surgery followed by hyperthermic intraperitoneal chemotherapy (HIPEC) for residual disease has been the mainstay of therapy for PMP. The purpose of surgical cytoreduction is not cure but palliative removal of the majority of tumor burden and resultant symptomatic improvement. Even with widespread peritoneal disease, the standard of treatment is repeated surgical debulking of symptomatic disease.[7,8] Survival is significantly dependent on completeness of

cytoreduction score (CCS).[7] Post-surgical 5-year overall survival range from 52-75%, the 10-year survival rates are low in the range of 10-32% only.[9-11]

Cytoreductive surgery followed by HIPEC is a protracted and strenuous procedure and results in significant morbidity and mortality. Therefore, only the best surgical candidates with little co-morbidity and an Eastern Cooperative Oncology Group (ECOG) performance status (PS) of 0-1 can undergo this procedure. It allows delivery of high doses of chemotherapy directly to the peritoneum (intraperitoneal heated (41°celsius) chemotherapy has enhanced cytotoxicity and better penetration). Still the majority of patients eventually recur and multiple efforts to repeat cytoreductive surgeries are done. However, repeat cytoreductive surgery is often not possible due to disease extent or complications from prior cytoreductive surgeries.[8,12] Currently, outside of surgical debulking and HIPEC therapy, supportive care is commonly recommended and no other therapy is considered a standard of care.

1.3.2 Systemic Chemotherapy

The role of systemic chemotherapy has been evaluated by a limited number of small retrospective studies and one phase II trial. A retrospective analysis of patients with disseminated appendiceal neoplasm who were not considered optimal candidates for cytoreductive surgery (n = 54) and received 2 or more cycles of systemic chemotherapy demonstrated a 55.6% disease control rate with median progression-free survival and overall survival of 7.6 months and 56 months, respectively.[13] Another retrospective analysis of metastatic poorly differentiated/signet ring cell appendiceal adenocarcinomas (n = 78) showed that chemotherapy lead to a radiographic response in 44% patients, a median progression-free survival (PFS) of 6.9 months and a median overall survival (OS) of 1.7 years. This analysis showed that systemic chemotherapy appears to be a viable and efficacious treatment option for patients with high-grade metastatic appendiceal adenocarcinomas.[14] However, high-grade appendiceal adenocarcinomas (moderate or poorly differentiated) frequently do not have mucinous disease and have a unique biology and thus, this population will not

be a part of this study. A phase II study evaluating combination of systemic concurrent mitomycin C and capecitabine in patients with advanced unresectable PMP (n = 40) showed that 38% (95% confidence interval: 25 to 54%) benefited from chemotherapy in the form of either reductions in mucinous deposition or stabilization of progressive pretreatment disease.[15] However, this study only used semiquantitative assessments and did not provide any information regarding the duration over which progressive disease was defined prior to the start of this study. Frequently disease appears stable over short-interval scanning (e.g. 3 months) but over a long period progression is seen. In addition this study enrolled primarily DPAM patients as this histological subtype represented 68% of the study population.

However, conclusive data about efficacy of these additional treatments including chemotherapy is deficient. Although, long progression-free survivals for PMP have been shown in retrospective studies of chemotherapy, whether this reflects the inherent disease biology or an impact from treatment is not known. At present for individuals that may not be optimal surgical candidates due to existing comorbidities or for which the tumor burden is so extensive that surgical cytoreduction is not feasible no standard treatment options exist besides supportive care, and thus it is critical to conduct a well-designed clinical trial to attempt to objective determine the potential benefit and role of systemic chemotherapy in this unusual disease type.

1.4 Measurements of treatment efficacy in PMP

There are other challenges in studying PMP. The mucinous peritoneal disease is hard to measure as it frequently exists and a contiguous erratically shaped area in the peritoneal cavity. As current RECIST criteria do not consider mucinous/cystic disease as measurable, standard RECIST criteria are not applicable. In addition this is a slowly progressive disease and classically defined changes in disease extent, such as a 20% increase, may take years to occur due to the natural history of this disease. Correlation between radiographic changes and intraoperative findings have been correlated with one

study of neoadjuvant chemotherapy prior to cytoreductive surgery showed stable disease on CT scans in 65% of pts while the intraoperative assessment showed stable disease in 50%.[16] Thus, determining systemic chemotherapy benefit, through standard outcome measures such as response rate and time to disease progression, cannot be determined in this tumor type. This study will thus utilize a novel quantitative measuring system designed for mucinous peritoneal disease. As the peritoneum is the only location involved by this disease process our measuring system, termed mucinous peritoneal RECIST (mpRECIST), will measure up to 5 areas of mucinous disease in the abdominal cavity. From unpublished retrospective data from 10 patients the use of mpRECIST demonstrated a 1.6% growth for patients on treatment over 6months and a 9% growth for a different cohort of patients on surveillance. The preliminary data suggests an effect size difference of 7.4% in tumor growth between treatment and non-treatment cases.

In addition as secondary endpoints this study will look at the indirect measures such as quality of life, semi quantitative tumor change, and change in tumor markers. The prospective QOL analysis will be performed using the generic QOL instrument, EORTC QLQ-C30 and the ovarian cancer specific subscale instrument, EORTC QLQ - OV 28. Since there is considerable similarity in symptomatology of peritoneal disseminated disease from ovarian cancer and PMP, we believe the QLQ-OV 28 is most appropriate for our patient population. A previous prospective study conducted at MD Anderson by our group in 50 patients has demonstrated the utility of these QOL measures for metastatic unresectable appendiceal adenocarcinomas (Abstract submitted to ASCO Gastrointestinal Symposium 2013).

1.5 Study Rationale

A large percentage of patients either fail primary cytoreductive surgery (CRS) or are unable to undergo surgery for a variety of reasons. Additionally, disease recurrence requires repeated and progressively more difficult surgery due to

adhesions and fibrosis and therefore after repeated CRS, eventually patients become non-surgical candidates. Management of these patients is challenging.

Due to the rare incidence of this malignancy, randomized clinical trials have not been performed evaluating the efficacy of chemotherapy in PMP. In absence of any prospective studies, there are a lot of unanswered questions. The pivotal question is whether systemic chemotherapy has a role to play in management of patients who have either failed CRS or are not surgical candidates. Although, retrospective data from small groups demonstrates prolong PFS times with systemic chemotherapy, the indolent natural history of PMP and inherent retrospective bias in these studies, makes it hard to determine treatment effect from inherent biological behavior.

There is no existing randomized clinical trial for this patient population to evaluate the benefit of chemotherapy in the world. The Phase II trial by Farquharson et al. was a nonrandomized Phase II study and lacks observation as a very critical control group.[15] Given the insufficient prospective data regarding the use of chemotherapy and our clinical experience, we have opted to assess potential clinical benefit of systemic treatment. The current trial design will provided needed evidence regarding the impact of chemotherapy. In particular the benefits of this study’s trial design are: (1) randomization of patients will minimize the bias from patient selection and (2) a cross-over design will enable each patient to serve as their own control and allow intra-patient comparisons that will be better able to answer the question of whether chemotherapy is a benefit for appendix PMP. The purpose of this prospective study is to evaluate the impact of chemotherapy on a novel quantitative assessment of mucinous peritoneal disease form PMP.
Performing a randomized controlled trial will help in answering this question and will help us better understand the biology of this disease. This will also help guide future research/treatment efforts. Furthermore, the results of this trial may help us develop an alternative treatment strategy for patients with PMP who are not candidates for CRS.

2 Objectives

2.1 Primary Objectives

- Difference in the percent change in mpRECIST between treatment and observation arms

2.2 Exploratory Objectives

- Comparison of QOL between treatment and observation periods
- Comparison of the percent change in tumor markers between treatment and observation periods
- Comparison of the semiquantitative response assessment by treating physician between treatment and observation periods
- Comparison of the rate of bowel complications defined as bowel obstruction requiring hospitalization or bowel perforation between treatment and observation periods
- Comparison of QOL between early and delayed chemotherapy approaches
- Comparison of OS between early and delayed chemotherapy approaches
- Comparison of percentage change in mpRECIST between blinded reviewer and treating physician
- Determining the rate of study dropout and reasons for dropout
- Exploratory analysis of the differing chemotherapy combinations with efficacy endpoints
- Exploratory analysis of volumetric analysis of mucinous peritoneal disease using image analysis software (Amira software version 5.4.2 by Visage Imaging)

3 Patient Eligibility

3.1.1 Inclusion Criterion:

i. Patients must have histological evidence of a metastatic well differentiated or moderately differentiated mucinous appendiceal epithelial neoplasm (AEN).

ii. Radiographic images demonstrating the presence of mucinous peritoneal carcinomatosis (PMP)

iii. Patients must not be considered a candidate for a complete surgical cytoreductive surgery. This determination will be made through either discussion at MD Anderson peritoneal surface malignancy multidisciplinary review or consultation with MD Anderson peritoneal surgeon.

iv. ECOG Performance Status 0-2.

v. Age $\geq$ 18 years old.

vi. Patients must be able to understand and provide answers to the EORTC QLQ-C30/OV-28 QOL questionnaires in order to participate in the trial.

vii. Adequate bone marrow function as evidenced by: Hemoglobin ≥9.0 g/dl; Platelet ≥75,000 cells/mm^3; Absolute neutrophil count ≥1000/mm^3.

viii. Women must not be pregnant or lactating. Women of childbearing potential must have a negative Beta-HCG serum pregnancy test and to refrain from breast-feeding, as specified in the informed consent given the unknown risk of teratogenicity of agents in the study. Patients of childbearing potential agree to use an effective form of contraception during chemotherapy and for 90 days following the last chemotherapy treatment.

ix. Patients must agree to participate and be able to understand and provide informed consent to participate in the trial.

3.1.2 Exclusion Criterion:

i. Concurrent uncontrolled medical illness that is deemed by the investigator to have potential to interfere with the delivery of chemotherapy for a six month time period.

ii. Patients who are receiving concurrent investigational therapy or who have received investigational therapy within 30 days of the first scheduled day of protocol treatment.

iii. The presence of complete or partial bowel obstruction based upon clinical assessment.

iv. Ongoing use of total parental nutrition

v. The presence of a concurrent non-appendiceal metastatic cancer.

4 Study Design

4.1 Treatment Plan

All surgically unresectable PMP/Well-differentiated mucinous appendiceal adenocarcinoma patients will be randomized to either Observation arm or Chemotherapy arm for 6 months and will then cross-over to the other arm at the end of this period (Figure 2). Each person serves as their own control in the study. Randomization will be performed through CORE.

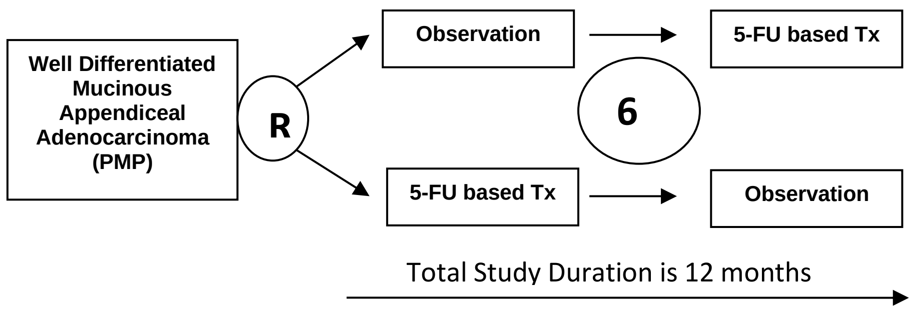

4.2 Determination of unresectability

Patients referred to or evaluated at The University of Texas MD Anderson Cancer Center (MDACC) with histologically confirmed well-differentiated mucinous appendiceal adenocarcinoma with radiographic evidence of mucinous peritoneal carcinomatosis (PMP) that are not surgical cytoreductive candidates are the eligible population for this study. Surgical candidacy will be determined by consultation with a MD Anderson surgeon or by discussion at Multidisciplinary Peritoneal Surface Malignancy Service at MDACC. Potential reasons for determining that a patient has unresectable disease could be: (1) the patient is not surgical candidate due to other co-morbidities, or (2) tumor bulk, such as encasement of the liver hilum or extensive small bowel involvement precludes the possibility of performing a complete cytoreductive surgery, or (3) a prior cytoreductive surgery has been unsuccessful. A complete cytoreductive surgery is defined as the ability to achieved a CC-0 (no-visible disease remaining) or CC-1 (greatest size of visible disease remaining is <2.5mm) resection.

4.3 Chemotherapy treatment

As discussed in the background section no standard systemic chemotherapy treatment exists for appendiceal cancers. Only one phase II study has been conducted in this disease type and this used a regiment of capecitabine and mitomycin.[15] The chemotherapy regimens used in colorectal cancer are used to treat appendiceal cancer. As all standard regimens for colorectal cancer utilize a fluoropyrimidine then current standard of care regarding systemic chemotherapy for this disease type would be a fluoropyrimidine-based treatment. NCCN clinical guidelines currently list as options for metastatic colorectal cancer as: FOLFOX +/-bev, CAPOX +/-bev, FOLFOX+/-panitumumab, FOLFIRI+/-bev, FOLFIRI+/-cetuximab or panitumumab, 5-FU +/-bev, capecitabine +/-bev, and FOLFOXIRI. In absence of any standard of care for such tumors, the study design recommends use of fluoropyrimidine based chemotherapy due to the past experience with this therapy in PMP and the

extrapolations of chemotherapy that are drawn from how we treat colorectal cancer.[14,15] Recommendations for chemotherapy will be provided by MD Anderson treating physicians according to the following prioritization sequence:

1. FOLFOX or CAPOX with Bevacizumab
2. FOLFOX or CAPOX without Bevacizumab
3. 5FU or Capecitabine with Bevacizumab
4. 5FU or Capecitabine without Bevacizumab
5. FOLFIRI with Bevacizumab
6. FOLFIRI without Bevacizumab
7. FOLFIRI with Cetuximab or Panitumumab (Kras wildtype only)
8. FOLFOX with Panitumumab
9. FOLFOXIRI

The exact type of fluoropyrimidine-based chemotherapy is not mandated and final treatment decisions will be left to the medical oncologist who is administering the chemotherapy. All chemotherapy adjustments will be done by the treating medical oncologist according to standard of care practice.

4.4 Supportive Care

At all times patients will receive maximal supportive care treatments for cancer-related symptoms. As bowel dysfunction is a common problem in this cancer these therapies may include the recommendations to eat multiple small meals a day or the use metoclopramide to help with gastrointestinal motility. If a bowel obstruction should occur than patients will be treated according to standard of care practice. Such treatment may include bowel rest, intravenous fluids, surgical consultation, venting G-tube, or total parental nutrition.

5. Evaluations During Study

Table 1: Evaluations During Study

**Evaluation or Procedure**	**Screening[1]**	**Study Treatment**	**End of Treatment Evaluation**	**Long Term Overall Survival Follow-up**
		Every 3 months +/- 2wks	12months +/-4weeks	
Informed consent	X			
Medical History	X			
Physical Examination	X	X	X	
Inclusion/Exclusion criteria	X			
Vital Signs (Blood Pressure)	X	X	X	
ECOG Performance Status	X	X	X	
Hematology[5]	X	X	X	
Biochemistry[5]	X	X	X	
Serum or urine pregnancy test (for females of child-bearing potential)	X			
Tumor Tissue Collection	X[2]			
QOL assessment[6]	X	X	X	
Diagnostic Imaging for Tumor Assessment[7]	X	X	X[3]	
MUIS assessment[8]	X		X	
Survival				X[4]

1. Screening studies must be completed within 2 weeks of signing consent except for radiographic studies which can be within 4 weeks of signing consent.
2. Archival tissue will be collected and stored as per institutional Tissue Station requirements. Patients may initiate study treatment prior to the receipt of the archival tissue. The collection of tumor tissue is not an absolute requirement for study

enrollment and if no prior tissue is available, then patients may still be enrolled and undergo study treatment.

3. The end of treatment imaging does not have to be repeated if recent imaging has been completed within 4 weeks.
4. Patients will be followed for overall survival/death once per year.
5. All laboratory tests will be conducted as standard of care according to each treating physician and should generally include a complete blood count, electrolytes, and liver function tests (AST, ALT, Alkaline phosphatase, Total bilirubin). Tumor markers (CEA, CA19-9 and CA-125) will be done at baseline and elevated markers will be followed at each subsequent approximate 3 month visit.
6. Quality of life will be assessed using the generic QOL instrument, EORTC QLQ-C30 and the ovarian cancer specific subscale instrument, EORTC QLQ - OV 28, and the anxiety specific scale of Speilberger State/Trait Anxiety Inventory State (STAI) at baseline, and every 3 months (+/- 2 weeks). The EORTC QLQ-C30 assesses the quality of life of cancer patients, the EORTC QLQ-OV28 assesses symptoms and side effects related to ovarian cancer, and the STAI assesses patients' anxiety. As question #58 on the EORTC QLQ-OV28 pertains only to female patients, male patients will be instructed not to answer this question and responses to this question will not be analyzed for male patients. The questionnaire will be given to the patient to complete at the visit and will be collected by the research staff in the clinic for each assessment. If the patient cannot complete the questionnaire before the end of the visit, they will be provided with a stamped, self-addressed envelope so that they can return the questionnaire by mail.
7. A CT scan (or MRI) of the abdomen and pelvis will be performed at baseline, and every 3 months (+/- 2 weeks) as standard of care. Contrast enhanced imaging is strongly recommended. The modality and need for imaging of the chest will be determined by each treating physician. Standard RECIST evaluation will not be applicable due to disease characteristics. We will therefore use a modified peritoneal RECIST (mpRECIST) that has been utilized at MD Anderson to assess quantitative changes in mucinous peritoneal disease.

8. The Mishel Uncertainty in Illness Scale (MUIS) assesses patients' perceptions of uncertainty about their symptoms, diagnoses, treatment, prognosis, and relationship with caregivers. It will be given at the start and end of the study. The questionnaire will be given to the patient to complete at the visit and will be collected by the research staff in the clinic for each assessment. If the patient cannot complete the questionnaire before the end of the visit, they will be provided with a stamped, self-addressed envelope so that they can return the questionnaire by mail.

6. Criteria for Removal from the Study
   a. Request of the patient for any reason
   b. If in the opinion of the investigator, it was in the patient's best interest to do so.
   c. Clinical deterioration from progressive disease.
   d. Uncontrollable toxicity
   e. Bowel complication (obstruction or perforation) requiring surgical intervention
   f. If tumor growth as determined by mpRECIST over a 3month interval is greater than 50%.

7. Response Criteria

Quantitative changes in mucinous peritoneal disease will be measured using the modified peritoneal RECIST (response evaluation criteria in solid tumours). The general guidelines for tumor evaluation will follow the structure established by RECIST version 1.1 except for the two fundamental differences: (1) up to 5 lesions in the peritoneal cavity will be assessed and (2) mucinous lesions will be considered measurable disease.[17] Up to five areas of mucinous peritoneal involvement will be measured at each evaluation. These lesions will be termed the target lesions. Measurable lesions are defined as those that can be accurately measured in at least one dimension (longest diameter to be recorded)

as >10 mm with CT scan or MRI scan (with minimum slice thickness no greater than 5mm. All tumor measurements must be recorded in millimeters (or decimal fractions of centimeters). Target lesions should be selected on the basis of their size (lesions with the longest diameter) and their suitability for accurate repeated measurements. In particular recommended sites for measurements are:

1. Any discrete isolated location
2. Thickness externally to liver at the level at which the main portal vein splits into the right and left portal vein
3. Thickness within the porta hepatis
4. Disease within the lesser sac
5. Thickness of omental disease
6. Thickness of disease at the level of the umbilicus
7. Thickness at the level of S1 nerve root

A sum of the longest diameters for all target lesions will be calculated and reported as the baseline sum diameters (LD). The baseline sum diameters will be used as reference by which to characterize the objective tumor measurement The standard RECIST criteria use changes in sum of 20% increase for progressive disease, and 30% shrinkage for a partial response, with stable disease occurring in between these two. However, as PMP is an indolent disease process these criteria will not be utilized in this study. In a pilot study of 10 patients the largest change in mpRECIST over a 6month period was 11%. Thus, the use of mpRECIST sum will be used in a comparative manner between treatment and non-treatment arms.

8. Statistical Methods

<u>Sample Size</u>

Based on preliminary retrospective data collected from two readers calculating mpRECIST on 5 patients the mean change over a six month time period on various common colon cancer treatments is an increase of 1.6% and without treatment the increase is 9%. This provides a 7.4% effect size with standard

error of 2.242%, so the 95% Confidence Interval (CI) is [2.95%, 11.74%]. The standard deviation of residuals is 3.47% for the random effects introduced by the two readers. If we consider both the variation introduced by different readers and variation of the treatment effects, the combined standard deviation of differences will be 4.135%. We have deemed a ≥5% difference in mpRECIST between observation and treatment periods (mpRECIST percent change on observation minus mpRECIST percent change on treatment) to represent a clinical meaningful difference between systemic chemotherapy treatment and observation. Thus, if we expect an 8% tumor difference between the two groups (Ha) and the sample size in each sequence group is 12 (a total sample size of 24), a 2 x 2 crossover design will 80% power to reject the null hypothesis that tumor shrinkage difference between control group and treatment group is less than or equal to 5% (Ho), assuming that the Crossover ANOVA square root of MSE is 4.0% (a rounded estimate from preliminary data) and using a crossover ANOVA with a 0.05 one-sided significance level.

As this is a cross-over design study over 12 months we anticipate that up to 20% of patients will not complete the full study period and thus plan to enroll up to 30 patients on this study to account for early drop-out or removal from the study. Patients who do not complete the entire 12 month study period will not be included in the primary efficacy analysis, but will be included in secondary analyses and adverse event analysis.

Efficacy Analysis

The primary objective is to determine if the difference in tumor growth rate for each patient, as measured by mpRECIST, between observation and systemic chemotherapy periods is ≥5%. The growth rate will be determined as the percent reduction from the start of the period to the 6 month measurement in that period. Crossover analyses will be performed according to Senn's methods (2002). In brief, first a test for period effect will be performed to determine whether similar treatments can be combined for a test of observation vs. treatment. If not, an ANOVA of treatment and order will be implemented

with an interaction for treatment and order for patients with complete information. Patients who were replaced due to missing efficacy information will be included in a secondary analysis, with missingness handled similarly to QOL, as described below.

Secondary objectives are to evaluate change in QOL, tumor markers, semiquantitative tumor changes, volumetric computer aided image analysis of peritoneal mucinous disease, and rate of death/bowel complications between treatment and observation periods. In addition secondary objectives are to compare OS and QOL between the early and delayed treatment arms.

Descriptive statistics, such as mean, standard deviation and median will be used to summarize the function scale, symptom scale, and the global health status scale of QOL. Paired student's t-test will be carried out to assess the difference in QOL between baseline and 6 months and 6months and 12 months. For patients who couldn't complete a survey, every effort will be taken to record the reason. We will also compare the baseline patient characteristics, baseline QOL scores and change between baseline and 3 month, and 6 month and 9 month in QOL scores, between the patients who could versus who could not complete the 6 or 12 month evaluation. A multiple logistic regression model will be fit for the indicator of "missing 6-month data" or "missing 12-month data", using patients' baseline characteristics and previous (baseline and 3-month or 6 month and 9 month) QOL scores as predictors. This model will help us to identify the factors that may have influenced patients' tendency of dropout. In addition to the primary analysis for the change of QOL between baseline and 6 month, and 6 month and 12 month, a linear mixed effect model will be fit for all patients to assess the change of QOL over time, where baseline QOL and time will be fitted as covariates (fixed effects) and patient effect will be included as a random effect. In order to avoid bias due to potential informative drop-outs (e.g., the patients with rapidly growing disease are more likely to drop out of the study), we will apply the pattern mixture model (PMM) (Little & Rubin, 1987) for

our data, which will include the patterns of missingness as a factor in the model. Sensitivity analysis could also be carried out to assess the robustness of our inference when different missing mechanisms are assumed, i.e., MAR (missing at random), MCAR (missing complete at random) and ID (informative dropouts).

For secondary analyses, semiquantitative response will be determined by the treating physicians, and for those with elevated tumor markers, the change in tumor markers will be determined with the 95% confidence intervals. The overall survival (OS) will be estimated using the Kaplan-Meier method for each study arm. The median OS will be calculated, along with the 95% confidence intervals.

Adverse Event Monitoring

The rate of adverse events will be monitored by a composite of death and bowel complications (defined as bowel perforation or hospitalization for bowel obstruction). This composite will be monitored in each arm. As bowel complications are reflective of the severity of progressive disease this monitoring will ensure that no unexpected differences in adverse events from progressive cancer are being observed between the two treatment arms. During the study, adverse events will be continuously monitored according to the table below, which was created by calculating a Fisher's exact test for each combination of possible numbers on each arm. When the adverse events in either group are significantly greater than the other group, we shall close the study early. Since the two treatments are observation and standard chemo, there are no rules for stopping based on individual adverse events rates.

Table 2: Early termination rules based on sample size and adverse events for a significant difference between the two groups.

N=12

**Higher**	If there are this many **adverse events**	3	4	3 or 4	5	5	6	5 or 6	7	7 or 8	7 or 8	9
**Rate**	**AND** this many **evaluable patients**	3	4	5	5	6	6	7	7	8	9	9
**Lower**	**Then stop** if there are this many **or *fewer* adverse events**	1	1	0	2	0	1	0	2	1	0	1
**Rate**	And this many **evaluable patients**	9	8	7	7	6	6	5	5	4	3	3

N=18

**Higher**	If there are this many **adverse events**	3	4	5	6	4	5	6	7	4	5	6	7	8	5	6 or 7	8	9
**Rate**	**AND** this many **evaluable patients**	6	6	6	6	7	7	7	7	8	8	8	8	8	9	9	9	9
**Lower Rate**	**Then stop** if there are this many **or *fewer* adverse events**	0	1	2	5	1	2	3	5	0	1	2	3	5	0	1	3	4
	And this many **evaluable patients**	12	12	12	12	11	11	11	11	10	10	10	10	10	9	9	9	9

**Higher**	If there are this many **adverse events**	5 or 6	7 or 8	9	10	6 or 7	8	9	10	11	7or 8	9-10	11	12
**Rate**	**AND** this many **evaluable patients**	10	10	10	10	11	11	11	11	11	12	12	12	12
**Lower Rate**	**Then stop** if there are this many **or *fewer* adverse events**	0	1	3	4	0	1	2	3	4	0	1	2	3
	And this many **evaluable patients**	8	8	8	8	7	7	7	7	7	6	6	6	6

9. Data and Protocol Management

Patients will be entered and randomized equally in Clinical Oncology Research System (CORE) and data will be collected in an excel database by the research team assigned to the study and all computer files will be password-protected and stored on institution computers behind the institution firewall to further ensure database security and all records are kept confidential. Patients will be seen by the research team at baseline and at each 3 month follow-up visit for the 12 month duration of the study. Following the completion of the 12 month study, patients will only be followed once per year for overall survival/death.

As this is a primarily an observational using known chemotherapy that has been extensively studied in colorectal cancer, chemotherapy related adverse events will not be captured. Only selected adverse events that will be monitored in our toxicity monitoring will be collected at each evaluation time-point and also patients will be instructed to contact the research nurse if any unplanned hospitalization occurs. From data collected at every 3 month (+/-2 week) evaluation and from data collected from unplanned hospitalizations the research team will follow toxicities relating to potential signs of peritoneal progression as discussed under the toxicity monitoring section.

Participant data will be kept confidential. When the questionnaire packets are given to patients, the patient's name will appear on a removable cover sheet, and a study identification number will be placed on each page of the questionnaire. Before the patient returns the questionnaire he/she will remove the cover sheet so no identifying information appears on the questionnaires. The patients' names will be linked to the study identification number in a database that will be password protected, with access restricted to the research coordinator and principal investigators.

10. Correlative Studies

All patients who sign consent for this study will provide consent for the collection of previously collected paraffin embedded tissue. Up to 30 unstained slides of 5 to 10 microns thick will be requested from the outside institution or prepared from the patient's tumor block, if applicable. The large number of slides relates to the highly mucinous nature of this tumor type and low amount of actual tumor that will be present on each slide. The tissue will be stored at MD Anderson Cancer Center as per institutional Tissue Station requirements. Any residual tumor tissue above what is specified here will be returned to the sending institution. Collected paraffin embedded tissue will be handled by Surgical Oncology research personnel. Only the PIs and his authorized research staff will have access to the identifiable information from this study. All the samples extracted from the paraffin tissue used in this study will be coded and identifiers (name, medical record number) will not be present on any material that is undergoing analysis. Patient information and relevant data will be stored on password – protected institution computers behind the institution firewall. At the closure of this study all unused material will be either returned to the sending institution or destroyed.

The primary objective of the correlative studies is to identify the molecular abnormalities that occur in PMP. In particular we plan to identify common oncogenic DNA mutations in this cancer. As the technology for DNA sequencing is rapidly changing the exact platform used will depend on the most robust platform at the time of tumor tissue analysis.

11. References

1. McCusker, M.E., Cote, T.R., Clegg, L.X. & Sobin, L.H. Primary malignant neoplasms of the appendix: a population-based study from the surveillance, epidemiology and end-results program, 1973-1998. *Cancer* **94**, 3307-3312 (2002).
2. Connor, S.J., Hanna, G.B. & Frizelle, F.A. Appendiceal tumors: retrospective clinicopathologic analysis of appendiceal tumors from 7,970 appendectomies. *Dis Colon Rectum* **41**, 75-80 (1998).
3. Nitecki, S.S., Wolff, B.G., Schlinkert, R. & Sarr, M.G. The natural history of surgically treated primary adenocarcinoma of the appendix. *Ann Surg* **219**, 51-57 (1994).
4. Sugarbaker, P.H.*, et al.* Pseudomyxoma peritonei syndrome. *Adv Surg* **30**, 233-280 (1996).
5. Raghav, K.P., Taggart, M.W., Fournier, K.F. & Overman, M.J. Is malignant dedifferentiation for mucinous appendiceal neoplasms a valid phenomenon or merely histopathologic ambiguity? *J Am Coll Surg* **212**, 906; author reply 907 (2011).
6. Ronnett, B.M.*, et al.* Disseminated peritoneal adenomucinosis and peritoneal mucinous carcinomatosis. A clinicopathologic analysis of 109 cases with emphasis on distinguishing pathologic features, site of origin, prognosis, and relationship to "pseudomyxoma peritonei". *Am J Surg Pathol* **19**, 1390-1408 (1995).
7. Baratti, D.*, et al.* Pseudomyxoma peritonei: clinical pathological and biological prognostic factors in patients treated with cytoreductive surgery and hyperthermic intraperitoneal chemotherapy (HIPEC). *Ann Surg Oncol* **15**, 526-534 (2008).
8. Hinson, F.L. & Ambrose, N.S. Pseudomyxoma peritonei. *Br J Surg* **85**, 1332-1339 (1998).
9. Smith, J.W.*, et al.* Pseudomyxoma peritonei of appendiceal origin. The Memorial Sloan-Kettering Cancer Center experience. *Cancer* **70**, 396-401 (1992).
10. Gough, D.B.*, et al.* Pseudomyxoma peritonei. Long-term patient survival with an aggressive regional approach. *Ann Surg* **219**, 112-119 (1994).
11. Fernandez, R.N. & Daly, J.M. Pseudomyxoma peritonei. *Arch Surg* **115**, 409-414 (1980).
12. Sugarbaker, P.H. & Chang, D. Results of treatment of 385 patients with peritoneal surface spread of appendiceal malignancy. *Ann Surg Oncol* **6**, 727-731 (1999).
13. Shapiro, J.F.*, et al.* Modern systemic chemotherapy in surgically unresectable neoplasms of appendiceal origin: a single-institution experience. *Cancer* **116**, 316-322 (2010).
14. Lieu, C.H.*, et al.* Systemic chemotherapy and surgical cytoreduction for poorly differentiated and signet ring cell adenocarcinomas of the appendix. *Ann Oncol* **23**, 652-658 (2012).
15. Farquharson, A.L.*, et al.* A phase II study evaluating the use of concurrent mitomycin C and capecitabine in patients with advanced unresectable pseudomyxoma peritonei. *Br J Cancer* **99**, 591-596 (2008).
16. Sugarbaker, P.H., Bijelic, L., Chang, D. & Yoo, D. Neoadjuvant FOLFOX chemotherapy in 34 consecutive patients with mucinous peritoneal carcinomatosis of appendiceal origin. *J Surg Oncol* **102**, 576-581 (2010).
17. Eisenhauer, E.A.*, et al.* New response evaluation criteria in solid tumours: revised RECIST guideline (version 1.1). *Eur J Cancer* **45**, 228-247 (2009).
18. Senn, Stephen. Cross-over Trials in Clinical Research (Textbook, 2nd Edition) Wiley (2002)

Appendix A

**ECOG PERFORMANCE STATUS***	
**Grade**	**ECOG**
0	Fully active, able to carry on all pre-disease performance without restriction
1	Restricted in physically strenuous activity but ambulatory and able to carry out work of a light or sedentary nature, e.g., light house work, office work
2	Ambulatory and capable of all self-care but unable to carry out any work activities. Up and about more than 50% of waking hours
3	Capable of only limited self-care, confined to bed or chair more than 50% of waking hours
4	Completely disabled. Cannot carry on any self-care. Totally confined to bed or chair
5	Dead

* As published in Am. J. Clin. Oncol.: *Oken, M.M., Creech, R.H., Tormey, D.C., Horton, J., Davis, T.E., McFadden, E.T., Carbone, P.P.: Toxicity And Response Criteria Of The Eastern Cooperative Oncology Group. Am J Clin Oncol 5:649-655, 1982.*
