# Supplementary material for: Efficacy of Systemic Chemotherapy in Patients With Low-grade Mucinous Appendiceal Adenocarcinoma: A Randomized Crossover Trial
Source: JAMA Netw Open. 2023 Jun 1;6(6):e2316161. doi: 10.1001/jamanetworkopen.2023.16161 (PMC10236240; doi:10.1001/jamanetworkopen.2023.16161)
Supplement: Supplement 3. — Data Sharing Statement [file jamanetwopen-e2316161-s003.pdf]

Data Sharing Statement

Shen. Efficacy of Systemic Chemotherapy in Patients With Low-Grade Mucinous Appendiceal Adenocarcinoma. *JAMA Netw Open*. Published June 01, 2023.
doi:10.1001/jamanetworkopen.2023.16161

Data
Data available: Yes
Data types: Deidentified participant data, Participant data with identifiers, Data (not involving human participants), Data dictionary
How to access data: The authors confirm that the data supporting the findings of this study are available within the article [and/or] its supplementary materials. And any additional data are available on request from the corresponding author
When available: With publication

Supporting Documents
Document types: None

Additional Information
Who can access the data: researchers whose proposed use of the data has been approved
Types of analyses: for a specified purpose
Mechanisms of data availability: after approval of a proposal
